# Information and strategic voting

**DOI:** 10.1007/s10683-015-9443-2

**Published:** 2015-05-17

**Authors:** Marcelo Tyszler, Arthur Schram

**Affiliations:** 1 Royal Tropical Institute (KIT), Amsterdam, The Netherlands; 2 Amsterdam School of Economics, Center for Research in Experimental Economics and Political Decision Making (CREED), Roetersstraat 11, 1018 WB Amsterdam, The Netherlands

**Keywords:** Voting behavior, Experimental economics, Quantal response equilibrium

## Abstract

We theoretically and experimentally study voter behavior in a setting characterized by plurality rule and mandatory voting. Voters choose from three options. We are interested in the occurrence of strategic voting in an environment where Condorcet cycles may occur and focus on how information about the preference distribution affects strategic behavior. We also vary the relative importance of the second preferred option. Quantal response equilibrium analysis is used to analyze the game and derive predictions. Our results indeed show that strategic voting arises. Its extent depends on (i) information availability; (ii) the relative importance of the intermediate candidate; (iii) the electorate’s relative support for one’s preferred candidate; (iv) the relative position of the plurality-supported candidate in one’s preference ordering. Our results show that information serves as a coordination device where strategic voting does not harm the plurality-preferred candidate’s chances of winning.

**Electronic supplementary material:**

The online version of this article (doi:10.1007/s10683-015-9443-2) contains supplementary material, which is available to authorized users.

## Introduction

Since its introduction in ancient Greece, democracy has always been associated with ‘government by the people’. A widespread view is that the democratic decision process must honor the desire of the majority (Goldfinger 2004). Voting is the tool most often used for this purpose. The underlying assumption is that voting correctly aggregates individual preferences. A sufficient condition for correct aggregation is that every voter casts a vote for her most preferred alternative. Of course, not everyone does so. For one thing, many people abstain from voting (especially in large scale elections). Moreover, voters may *strategically* vote for an alternative that is not ranked highest in their preference ordering (Farquharson 1969). The reason is that any election is not only a manifestation of individual preferences, but also a multi-person decision process (Downs 1957; Riker 1982a; Blais and Nadeau 1996). In such a strategic interaction a voter may be more interested in optimizing the outcome than in stating her own preference.

In this paper, we investigate such strategic voting in a controlled (laboratory) environment. Our aim is to carefully isolate important determinants of the strategic vote. In particular, we are interested in the effect on strategic voting of information about others’ preferences and the relative attractiveness of the second-best alternative.

When considering voting as a multi-person decision process it can be analyzed as a strategic game in which distinct strategies might lead to different outcomes and equilibria can be computed. It has long been recognized that strategic voting may be an equilibrium strategy in committees (Austen-Smith and Banks 1996), legislatures (Riker 1982a) and even in large electorates (Palfrey 1989; Fey 1997). Of course, strategic voting equilibria may involve highly complex computations that go beyond the capabilities of most voters. Behaviorally, voters may rely on simple voting heuristics such as always voting sincerely for the most preferred alternative. In addition, some people may object morally to voting strategically (Lehtinen 2007). In the end, the question whether or not voters vote strategically is an empirical one.^1^

An example illustrates situations when strategic voting may occur. If the most preferred option does not stand a chance, a voter may vote for her second ranked option in an attempt to avoid even worse outcomes. Such behavior is consistent, for example, with Duverger’s law.^2^ A special case occurs when there is a Condorcet loser (i.e., an alternative that would lose any pairwise vote against any other alternative) supported by a minority while a majority is divided between two other alternatives (Gerber et al. 1988; Forsythe et al. 1993, 1996; Cox 1997; Myatt and Fisher 2002; Palfrey 2006). The majority can avoid a victory by the Condorcet loser if the supporters of one of the two majority alternatives votes strategically for the second most preferred option. Though our goal is to better understand the occurrence of strategic voting, we do not consider situations with a Condorcet loser.

Instead, we are interested in situations where there are Condorcet cycles. In our environment, each of three alternatives (denoted by *A*, *B*, and *C*) has a similar a priori chance of winning the election and each voter faces an a priori symmetric strategic problem. A cycle occurs because sincere voting can lead to any of the alternatives winning if they are voted on sequentially in pairwise votes. We will see below, that the occurrence of Condorcet cycles yields incentives to vote strategically when decisions are made by plurality rule.

We use laboratory experiments for our empirical analysis of strategic voting. Before doing so, we first model the situation as a strategic game. In particular, we will derive Quantal Response Equilibria (QRE) and use these to formulate behavioral predictions. QRE accurately predicts voter behavior in many environments (Goeree and Holt 2005; Levine and Palfrey 2007; Groβer and Schram 2010). It has the intuitive advantage that it allows for boundedly rational behavior while at the same time assuming that the error people make declines as the stakes become larger. Our QRE predictions will be tested using our experimental data.

Laboratory control will allow us to measure the impact of changes in the environment on the decision whether or not to vote strategically. Specifically, we are interested in the two circumstances mentioned above. First, we will study how the relative value attributed to the second preferred option affects voters’ decisions. This is important because, intuitively, voters are more likely to vote strategically when there is little to lose by having their second option chosen (Blais et al. 2001). Second, we will measure the impact of information about others’ preferences. This is important, because whether or not voters vote strategically may depend on how much they know about other voters’ preferences (Forsythe et al. 1993, 1996). Outside the laboratory opinion polls serve to provide such information, which may help voters to coordinate on an alternative and win the election. Voluntary preference revelation in polls may be strategic, however. In order to isolate the effect of information, we therefore opt for a situation in which an opinion poll truthfully reveals the electorate’s preferences (as in Großer and Schram 2010). Perfect information about the other voters’ preferences will in some of our treatments be made available before the election.^3^

With this information, the decision problem faced by each voter may be even more complex than without. This is because without information all voters face the same a priori situation if every preference ordering is equally likely. Assume for the case with information that supporters of the alternative with the largest support (we call this the ‘majoritarian candidate’) vote sincerely but comprise less than 50 % of the electorate. Which voters should then vote strategically? On the one hand, one may think that the supporters of the alternative with the lowest level of support have an incentive to vote strategically to increase their chances. On the other hand, voters for whom the majoritarian candidate is second best may decide to support this to ensure at least this second-best. Whether or not they do so may depend on the relative value they attribute to this option. We will address these issues theoretically and behaviorally in this paper.

When preferences are not revealed by polls, all voters face the same situation. The QRE prediction is then that all voters have the same probability of voting strategically and this probability increases with the value attributed to the intermediate option. This comparative static prediction is confirmed by our data. With information about the other voters’ preferences, the prediction depends on the number of others supporting the same alternative and this alternative’s rank (in terms of support) within the electorate. It also depends on the relative value attributed to the second most preferred alternative. The experimental results are largely in line with the QRE predictions. Two important conclusions for the scenario with information are that (i) a higher frequency of strategic voting is observed, the higher is the relative utility of a voter’s second most preferred option; (ii) there is coordination on the victory of the majoritarian candidate. All in all, our results show that strategic voting is an important phenomenon and follows a pattern that to a large extent can be rationalized using the boundedly rational framework offered by QRE.

The remainder of this paper is organized as follows: Sect. 2 presents theoretical analysis and equilibrium predictions. The experimental design is introduced in Sect. 3. Section 4 presents the results and Sect. 5 offers concluding remarks.

## The model

Each of *N* voters must choose from three alternatives, *A*, *B* and *C*. Each voter *i* = 1,…, *N* has a strict preference ordering over these alternatives and must cast exactly one vote. Plurality rule determines the winner, with ties broken by an equal probability random draw. The assumption of mandatory voting allows us to focus on the voting decision without needing to correct for the interaction with the turnout decision. Moreover, the mandatory rule makes strategic voting more salient, since voters are obliged to decide. Mandatory voting exists in many committees and legislators (Nitzan and Procaccia 1986). For national elections, only a minority of countries has mandatory voting (Gratschew 2001), though it is still prevalent in cer-tain regions, like Latin America. Note that even in the absence of mandatory voting many people may feel sufficient warm glow, social pressure or sense of civic duty to vote. We therefore expect our results to be relevant beyond the particular case of mandatory voting assumed here.

Voters are assumed to maximize (expected) utility, where a voter’s utility is determined by the rank of the elected alternative in her preference ordering. If her preferred, intermediate or least preferred alternative is elected she receives *u* ^*b*^ *, u* ^*m*^ or *u* ^*l*^ respectively. Without loss of generality we normalize by setting *u* ^*b*^ = 10 and *u* ^*l*^ = 1. Then, each voter’s preferences are characterized by *u* ^*m*^, the utility attributed to the intermediate option. Finally, we assume that utility is independent of individuals and options, i.e., *u* ^*m*^ is the same for every voter.^4^ Hence, only the ordering of the three options distinguishes voters from one another.

We further assume that before an election all voters’ preferences are determined randomly, independently of previous preferences and of other voter’s draws. The own preferences are revealed to the voter by nature before the election. The extent of information about other’s preferences is a variable in the model. The setting can be either *uninformed*, in which case voters (aside from their own preference ordering) know only the prior probability distribution of preferences, or *informed*, in which case they know the ex-post realized distribution of preferences for the election concerned. This variable is meant to capture the possible publication of (noiseless) pre-election polls, as described in the introduction.

An electorate is, therefore, characterized by the number of voters, the distribution of preferences, *u* ^*m*^, and the extent of pre-election information. We define *sincere* voting as a vote for the most preferred option. A *strategic* vote is defined as a vote for the second-ranked alternative in the preference ordering (as in Blais and Nadeau 1996; Blais et al. 2001; Cain 1978). The third option, voting for the least preferred option, will only be considered as noisy behavior, because it is a dominated strategy: there is no circumstance under which this could serve the purpose of expected utility maximization.

Because we are most interested in strategic voting caused by the environment and not so much in specific characteristics of the distinct options, we will focus on a game in which every voter has an a priori symmetric problem regardless of his/her preference ordering. We therefore restrict the possible preferences to {(*A, B, C*); (*B, C, A*); (*C, A, B*)}, in which the listed order represents the preference ordering. Preferences are independently and randomly drawn from this set with equal probability for each voter. These preferences will typically form a Condorcet cycle, potentially giving rise to strategic behavior. Moreover, there are no Condorcet losers in our setup. We define *N*_*ABC*_ as the number of voters with preference ordering (*A, B, C*) [i.e., u(*A*) = 10; u(*B*) = *u* ^*m*^; u(*C*) = 1], and similarly *N*_*BCA*_ and *N*_*CAB*_. Note that by construction *N*_*ABC*_ + *N*_*BCA*_ + *N*_*CAB*_ = *N*. Finally, we denote the election outcome by a vector *v* ≡ (*v*_*A*_ *, v*_*B*_ *, v*_*C*_) such that *v*_*A*_ + *v*_*B*_ + *v*_*C*_ = *N*, where *v*_*k*_ denotes the number of votes for option *k*.

### Equilibrium analysis

Typically, multiple Nash equilibria exist in voting games. Take, for example, a situation in which *N* = 3 *K* (*K* ≥ 3) and each preference ordering is equally represented (*K* voters each) while there is complete information. Then, all situations are Nash equilibria in which voters in exactly two groups vote sincerely and the voters in the remaining group all vote strategically. The election outcome would be, for example, *v* = (0, 2 *K*, *K*) and since no voter is pivotal, no one can benefit from deviating. Another Nash equilibrium is where only one group votes sincerely, with the other two voting strategically. Again, nobody is pivotal. Sincere voting by all may also be an equilibrium. Such voting behavior leads to an expected payoff of (*u* ^*b*^ + *u* ^*m*^ + *u* ^*l*^)/3 = (11 + *u* ^*m*^)/3. If there are equal numbers of voters for each preference ordering each voter is pivotal, however. Voting strategically will therefore tip the balance to the own second preferred option and yield payoff *u* ^*m*^. As long as *u* ^*m*^ ≤ (11 + *u* ^*m*^)/3, everyone voting sincerely is a Nash Equilibrium.^5^ To tackle the multiple equilibria problem one can employ an equilibrium selection device. The QRE approach adopted here has as a spinoff that it constitutes such a refinement in the sense that it selects specific Nash equilibria as a special case.

For a variety of political choice problems, QRE, and in particular the Multinomial Logit equilibrium (MLE) (McKelvey and Palfrey 1995) better predicts individual choices than Nash equilibrium (Goeree and Holt 2005). For example, it can account for the (seemingly irrational) high turnout rates in large-scale national elections, where Nash predicts unrealistically low turnout (Levine and Palfrey 2007).

#### Uninformed setting

Consider first the situation without information about other voters’ preferences. The voter knows only the prior distribution of probabilities, the electorate size, the value of the intermediate option and her own preference. Knowing her own preference she can update the probability distribution and use this to calculate the probability of being pivotal given others’ strategies. Subsequently, she can compute her expected payoff differences between voting sincerely, strategically or for the least preferred alternative.

This rather complicated computation is easiest understood by an example. Consider the case *N* = 12 (which is the electorate size used in our experiments). For a given voter, the most likely distributions among the other voters are (3, 4, 4), (4, 3, 4) and (4, 4, 3), where the first number indicates the number of other voters with the same preference, and the other two the number in the remaining groups. If she believes that all others are voting sincerely this voter considers herself to be pivotal in all three situations (in the first she can create a tie, in the latter two she can break a tie). In the first situation her sincere vote would create a three-way tie and voting strategically would give the victory to her second most preferred candidate. Voting sincerely may be profitable, depending on the value of the intermediate option.^6^ For the other two situations voting sincerely is always a best response, since the voter is decisive in favor of her most preferred candidate. Considering only these three situations voting sincerely would likely be a best response. In fact, considering all pivotal situations with their respective probabilities it can be shown that voting sincerely is more profitable than voting strategically. Thus, all players voting sincerely constitutes a Bayesian-Nash Equilibrium, regardless of the inter-mediate preference parameter *u* ^*m*^.

**Fig. 1:**
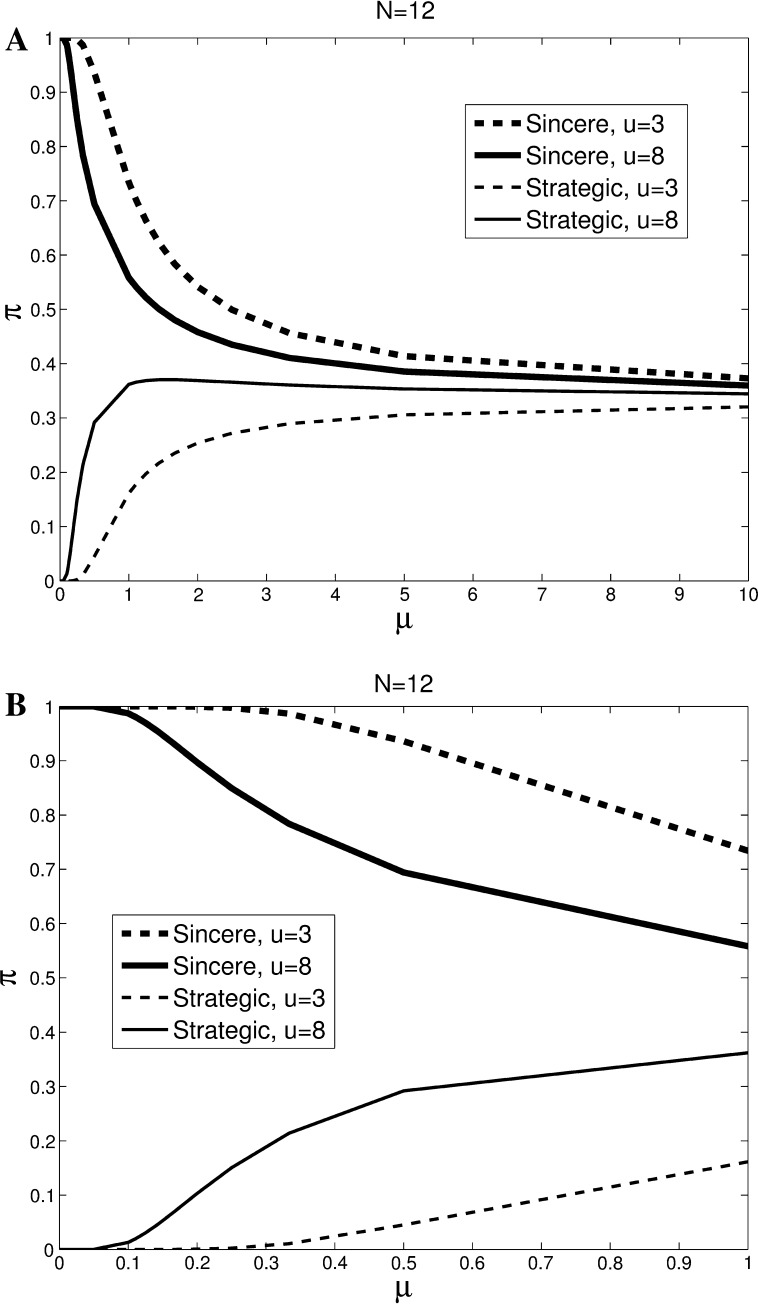
Multinomial logit correspondences for uninformed voters. *Lines* show the principle branch of the MLC for high (*u* ^*m*^ = 8) and low (*u* ^*m*^ = 3) values of the intermediate option. Panel **b** zooms in on *μ* ∈ [0,1]

As explained in online Appendix A, this Bayesian-Nash equilibrium is the limiting MLE of the game of incomplete information as the error parameter in this QRE (denoted by *μ*) approaches zero (McKelvey and Palfrey 1995). Figure 1 shows the corresponding Multinomial Logit Correspondences (MLC; this gives the set of MLE and corresponding error parameters) for voting bodies of size *N* = 12. See online Appendix E for corresponding graphs for larger voting bodies. We consider two values for the intermediate option: high (*u* ^*m*^ = 8) and low (*u* ^*m*^ = 3), which are the values that we use in our experiments.

Note that for *μ*↓0, the probability of sincere voting converges to 1. Hence, for the case of incomplete information (no polls) the limiting MLE is the Bayesian Nash equilibrium without strategic voting, irrespective of *u* ^*m*^. At the other extreme, when noise dominates behavior (*μ* → ∞), the vote becomes a random choice and voting sincerely, strategically or for the dominated option each occur with probability 1/3. For the intermediate cases where rationality is somewhat bounded [*μ* ∈ (0,∞)], the MLE probabilities of voting depend on the value attributed to the intermediate option. Previous estimates of *μ* using data from voting experiments yield values between 0.4 and 0.8.^7^ We will therefore focus some of our discussion on this range of *μ* values. As explained below, we will use for our specific predictions an out-of-sample estimation of the error parameter, which is *μ* = 0.55. This falls exactly within the range.

Note that the probabilities of voting for the distinct options strongly depend on both *μ* and *u* ^*m*^. First, it takes a high value of *μ* for voting for the dominated action (not shown) to be likely. When random noise does not dominate behavior (*μ* < 1) the probability of voting for the third option is less than 10 % and the choice is basically between voting sincerely or strategically. For u^m^ = 8, *μ* = 1, for example, the MLE probability of voting sincerely is 0.56 and the probability of voting strategically is 0.36. Hence, the probability of voting for the dominated option is 0.08. As for *u* ^*m*^, for *u* ^*m*^ = 8, the probability of voting strategically exceeds 0.25 for a wide range of *μ*-values. The intuition is that although the limiting (Bayesian Nash) equilibrium is to vote sincerely, one does not lose too much by choosing the second-best. Therefore, an ‘error’ to the best response is not very costly and more likely to occur in the MLE. Focusing on *μ* values between 0.4 and 0.8, the equilibrium probability of a strategic vote is more than three times as high for high intermediate utility than for *u* ^*m*^ = 3. For *u* ^*m*^ = 8 the model predicts that approximately 30 % of the voters will vote strategically for these *μ* values.

#### Informed setting

**Fig. 2:**
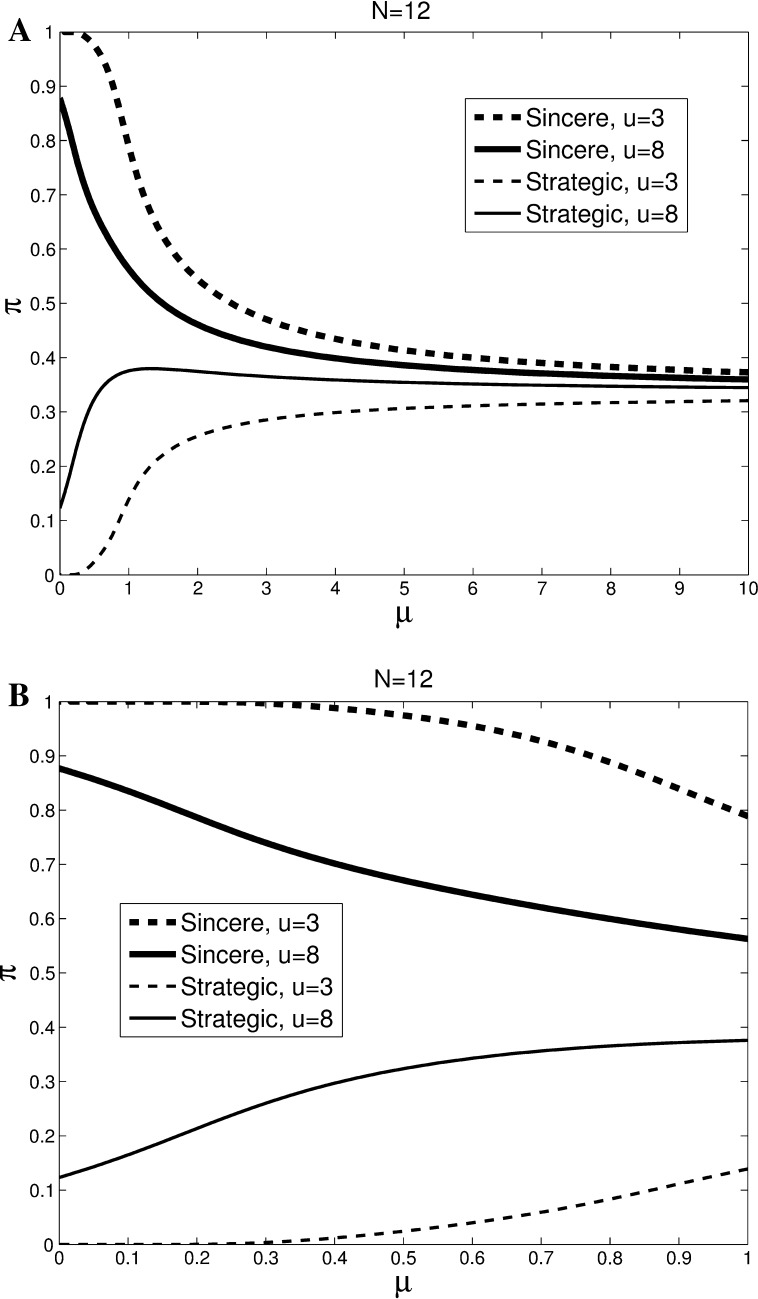
Multinomial logit correspondences for informed voters. *Lines* show the principle branch of the MLC for high (*u* ^*m*^ = 8) and low (*u* ^*m*^ = 3) values of the intermediate option. (*N*_*ABC*_ *, N*_*BCA*_ *, N*_*CAB*_) = (4, 4, 4). Panel **b** zooms in on *μ* ∈ [0,1]

Consider next the game with full information. Start with an example with equal share, which can serve as a comparison to the a priori expected situation for uninformed voters in Fig. 1. Figure 2 plots the principal branch of the MLC for small [(*N*_*ABC*_ *, N*_*BCA*_ *, N*_*CAB*_) = (4, 4, 4)] voting bodies (see online Appendix E for N = 99). In these cases all voters’ circumstances are again perfectly symmetric. In comparison to the previous case, however, information about others’ preferences removes the uncertainty. The Nash equilibrium of sincere voting is the limiting MLE when the inter-mediate option is relatively unattractive (*u* ^*m*^ = 3). For *u* ^*m*^ = 8, the probability of strategic voting converges to 0.12 as μ↓0 for *N* = 12. Hence, the MLE converges to mixed strategy Nash equilibria with (small) positive probabilities of voting strategically. Other results are quite similar to the uninformed case. The probabilities of voting for the dominated option are small for μ < 1 and large differences in strategic voting are predicted between *u* ^*m*^ = 8 and *u* ^*m*^ = 3 when *μ* ∈ [0.4, 0.8].

**Fig. 3:**
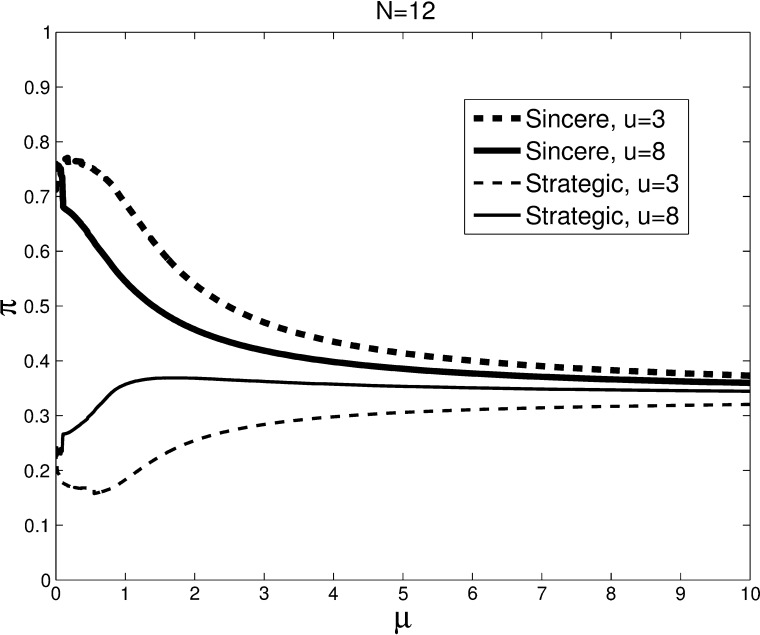
Average multinomial logit correspondences for informed voters. *Lines* show the weighted average of the principle branches of the MLCs for high (*u* ^*m*^ = 8) and low (*u* ^*m*^ = 3) values of the intermediate option. The average is across all possible combinations of preference orderings, weighted by the probabilities with which they occur

The equal split case is just one of the many distributions that may be realized (and revealed). In cases where the revealed distribution is unequal, one may expect patterns very different from the uninformed case of Fig. 1. Online appendix C shows the MLC graphs for all possible realizations in the *N* = 12 case. Online Appendix D provides for each realization the Nash equilibria selected by the limiting MLE. Figure 3 presents the weighted average of these MLCs, where the weights are given by the probabilities that specific realizations of the preference distribution will occur. Therefore, the equilibria in Fig. 3 represent average behavior across multiple committee votes with complete information.

Note that the average of the limiting Nash equilibria across preference configurations is not to vote sincerely. The limiting MLE predicts a weighted average of 73 %/76 % sincere voting and 24 %/22 % strategic voting for low and high intermediate value, respectively. Starting with very small *μ*, the roles are reversed: the MLE predicts more strategic voting when the intermediate value is high. Large differences in strategic voting are predicted between *u* ^*m*^ = 8 and *u* ^*m*^ = 3 when *μ* ∈ [0.4, 0.8].

Based on *μ* ∈ [0.4, 0.8], the analysis of the Principal Branch of the MLC yields a first set of behavioral predictions for *N* = 12, which we will test with our experimental data.

1. Without information, the probability of strategic voting is increasing in the importance of the intermediate option (Fig. 1).
2. With full information the probability of strategic voting is increasing in the importance of the intermediate option (Fig. 3).
3. When the value of the intermediate option is low, there is more strategic voting with information than without (Figs. 1 vs. 3).

In order to further structure the analysis, a few definitions are useful:

##### **Definition 1**

The *Majoritarian Set* is the set of alternatives with the highest number of votes if all voters vote sincerely.

##### **Definition 2**

The *Majoritarian Candidate* is the (set of) alternative(s) from the Majoritarian set with the highest number of votes if all voters vote sincerely for an alternative within the set.

If the Majoritarian Set is singleton it equals the Majoritarian Candidate. If it contains two elements (two options with equal sincere support, a third with less), then the Majoritarian Candidate is the option that gives highest utility to the supporters of the third option. The Majoritarian Candidate is unique, except when (*N*_*ABC*_ *, N*_*BCA*_ *, N*_*CAB*_) = (4, 4, 4).

For any distribution of preferences we now first classify voters based on the rank of their most preferred candidate.^8^

##### **Definition 3**

The *Rank*-*Type* of a voter is given by:

- *Rank 1st* Voter whose most preferred candidate is the Majoritarian Candidate.
- *Rank 2nd* Voter whose most preferred candidate is second in the (sincere) polls.
- *Rank 3rd* Voter whose most preferred candidate is third in the (sincere) polls.

By ‘sincere polls’ we mean the ranking that occurs if all voters vote sincerely. Duverger’s law suggests that the Rank 3rd voters will be most likely to vote strategically. However, the incentive to do so depends on the position of the Majoritarian Candidate in their preference ordering. For example, consider (*N*_*ABC*_ *, N*_*BCA*_ *, N*_*CAB*_) = (5, 4, 3). The Majoritarian Candidate is *A* and voters with preference ordering *CAB* are Rank 3rd. If voters with preference *ABC* vote sincerely, the Rank 3rd voters have no direct reason to vote strategically; their least preferred candidate will probably not win anyway. In contrast, Rank 2nd voters (preference *BCA*) may vote strategically in an attempt at a majority coalition with the Rank 3rd. Instead of the Rank-Type, the probability of strategic voting may therefore be determined by the benefits that the Majoritarian candidate gives to other voters than Rank 1st. We therefore define:

##### **Definition 4**

The *Incentive*-*Type* of a voter is given by:

- *Supporter* Voter with the Majoritarian Candidate as the most preferred alternative.
- *Compromiser* Voter with the Majoritarian Candidate as 2nd most preferred alternative.
- *Opposer* Voter with the Majoritarian Candidate as the least preferred alternative.

Note that the Rank 1st and Supporters are by construction the same group, but Rank 2nd (Rank 3rd) can be either Opposers (Compromisers) or Compromisers (Opposers). We can then identify four combination of Rank-Types and Incentives-Types other than Rank 1st. Online Appendix F presents and analyzes the equilibrium predictions for these combinations. Here, we use the analysis to derive an additional three behavioral predictions:

4. With full information Rank 3rd voters vote more strategically (on average) than other Rank-Types (Figure F1 in online Appendix F).
5. With full information and low value for the intermediate option, Opposers are more likely to vote strategically than Compromisers.
6. With full information and high value for the intermediate option, Compromisers are more likely to vote strategically than Opposers.

## Experimental design

12 sessions were run at the CREED laboratory at the University of Amsterdam, in November–December 2008. 288 student subjects participated in 24 independent electorates. Each session lasted about one and a half hours. In addition to a show-up fee of €7, subjects were paid €0.05 per experimental point. Average earnings were €20.46, including the show-up fee. The experiment was computerized using Z-tree (Fischbacher 2007). Instructions can be found in online Appendix G. The design aims at studying the impact on voting behavior of the relative importance of the intermediate option and the extent of information. A full 2 × 2 combinatorial design therefore requires four treatments. All variations were made across subjects.

The electorate is fixed during a session and consists of 12 voters. Each electorate faces 40 independent elections. In every election, there are three possible preference orderings, {(*A*, *B*, *C*); (*B*, *C*, *A*); (*C*, *A*, *B*)}, which are assigned with equal probability to each subject. There is a new draw before every election. Draws are independent across subjects and elections. Every individual is informed about his or her own preferences before each election. All this is common knowledge. Every experimental electorate experienced the same realization of the random draws (*cf*. online Appendix H), enabling a perfect comparison across electorates. Note that giving subjects a new preference ordering in each election has the advantage of providing us with a rich set of compositions of the electorate. It does reduce the learning effect, however.

In every election each subject is required to cast one vote for *A*, *B* or *C*. Plurality rule determines the winner, with ties broken by equal probability random draw. Subjects are paid in each round according to the rank of the winner in their own preference ordering. If the winner is the highest ranked option a subject is paid 10 points and for the lowest ranked 1 point. The value of the intermediate option is constant for a given electorate and is either 3 or 8 according to the treatment. In the informed treatments, participants know the aggregate induced preferences of all voters before casting their vote in a round. Specifically, they are told how many other voters where appointed to each of the three preference orderings. After each election, the aggregate voting outcome is shown to all subjects. Table 1 summarizes the design.

**Table 1:** Experimental design

Information	
Intermediate option	
Low importance (u = 3), uninformed	Low importance (u = 3), informed
High importance (u = 8), uninformed	High importance (u = 8), informed

For each cell, we have observations from six electorates. In addition, in August 2007, two pilot sessions were run at Fundação Getúlio Vargas, São Paulo, Brazil.^9^ We used data from this pilot to obtain an out-of-sample estimate of the MLE parameter *μ*, for which we found *μ* = 0.55. This provides us with specific MLE predictions for our experimental data.

## Results

We start with a general overview of voters’ choices and election outcomes in Sect. 4.1. Then, we study in more detail the occurrence of strategic voting across treatments in Sect. 4.2 and choices by distinct types in Sect. 4.3. In Sect. 4.4 we summarize our findings. Unless indicated otherwise, throughout this section our statistical tests will be non-parametric using average numbers per electorate as units of observation.

### General overview

For a first impression of the data, Table 2 shows for each treatment the distribution of votes across options. Because the labels *A*, *B*, and *C* have no real content, we aggregate votes for most preferred, intermediate, and least preferred option.

**Table 2:** Vote distribution

	Information					
	Uninformed			Informed		
Intermediate option						
*u* ^*m*^ = 3	1:0.936	2:0.049	3:0.015	1:0.806	2:0.169	3:0.025
*u* ^*m*^ = 8	1:0.798	2:0.192	3:0.010	1:0.740	2:0.245	3:0.015

Numbers give the fractions of voters in the treatment denoted by the combination of column and row that voted for, respectively, the (1:) most, (2:) intermediate and (3:) least preferred options

A first thing to note is that we very rarely see subjects voting for the dominated, least preferred option. Second, strategic voting (voting for the intermediate option) is highest (almost 25 %) when the intermediate value is high and subjects are informed about the preference distribution.^10^ A Kruskal–Wallis test shows that both the fraction of sincere voting and the fraction of strategic voting differ significantly across the four treatments (for sincere voting: *χ* ^*2*^ = 18.12, *p* < 0.01, *N* = 24; for strategic voting: *χ* ^*2*^ = 17.49, *p* < 0.01, *N* = 24). Pairwise comparisons follow below. This shows that the combination of information and the value attributed to a voter’s second-best candidate significantly affect the decision whether or not to vote strategically. We analyze the determinants of strategic voting in more details, below.

**Fig. 4:**
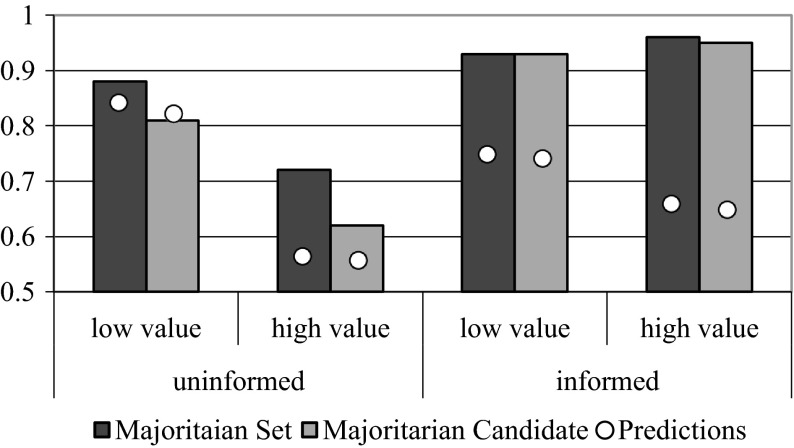
Majoritarian set and majoritarian candidate. *Bars* show for each treatment the fraction of election outcomes that are, respectively in the Majoritarian Set or equal to the Majoritarian Candidate. *Dots* denote the theoretical predictions

Before doing so, we consider the election outcome. In particular, Fig. 4 shows, across treatments, the fraction of elections where the winner was the Majoritarian Candidate or in the Majoritarian Set.^11^ This shows that without information both are better predictors of the election outcome when the intermediate value is low. This is in line with intuition because behavioral prediction 1 is that less strategic voting is to be expected when *u* ^*m*^ = *3* which in turn will improve the chances of the Majoritarian Candidate. Moreover, information leads to strong coordination around the Majoritarian Candidate, which wins over 93 % of the elections.

### Strategic voting

**Fig. 5:**
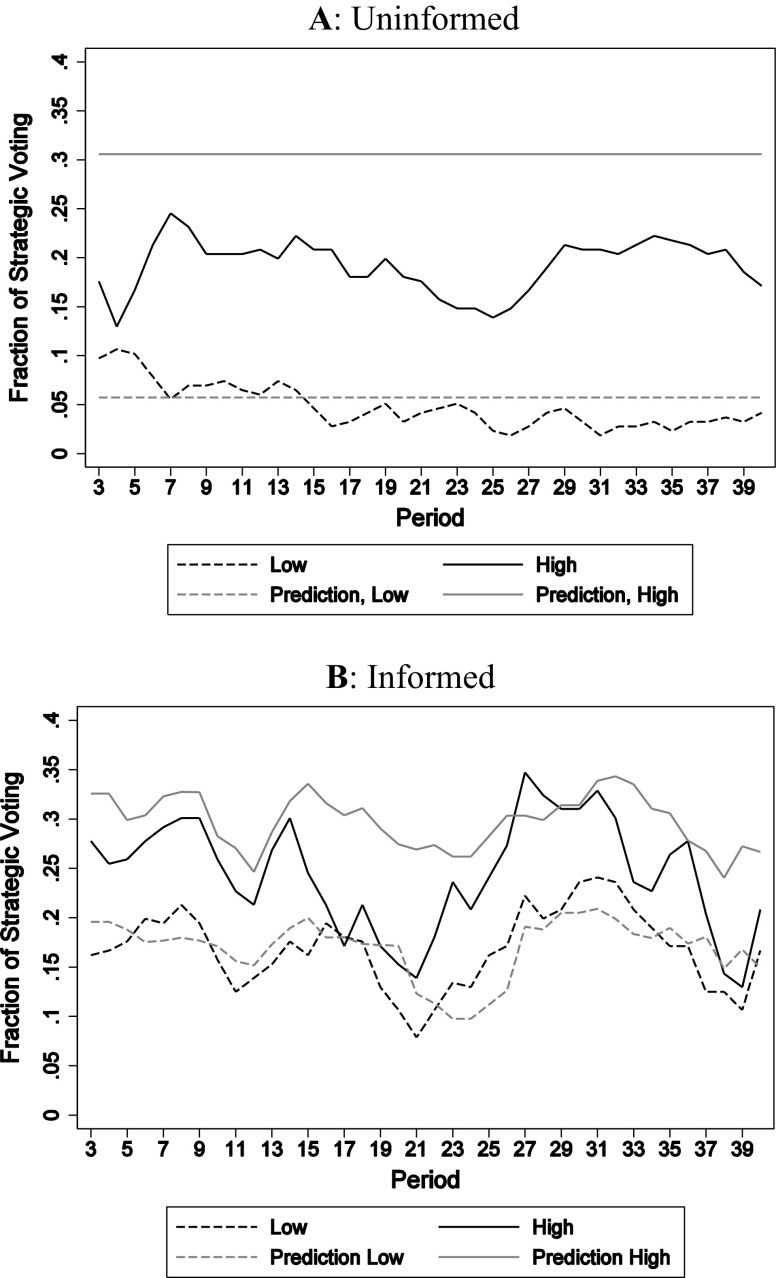
Experimental data and predictions*. Lines* show the 3-period moving average of the fraction of strategic votes in the uninformed (panel **a**) and informed (panel **b**) sessions. *Dashed* (*solid*) *lines* refer to low (high) intermediate values. *Light lines* show the 3-period moving average MLE predictions. Note that in the informed case (panel **b**) the MLE prediction in a round depends on the realized distribution of preferences

For each treatment Fig. 5 shows the fraction of strategic votes across rounds. It includes the MLE predictions based on the value for *μ* estimated with the pilot data (*cf*. Sect. 3). A first, general impression is that the MLE predictions for low intermediate values fare quite well. For both the uninformed and the informed cases, the data are close to the prediction. For high intermediate values, the observations appear to be somewhat lower than predicted.

Comparing across treatments, we observe more strategic voting when the intermediate value is high than when it is low. The difference is statistically significant for both informed and uninformed voters (in both cases, Mann–Whitney (*MW*), *Z* = −2.882, *p* < 0.01, *N* = 12). This is in support of behavioral predictions 1 and 2 of Sect. 2. In short, even though the observed extent of strategic voting when *u* ^*m*^ = 8 is somewhat lower than predicted by MLE, the comparative static prediction that it is higher than for *u* ^*m*^ = 3 finds (strong) support in our data.

A comparison of panels A and B shows whether information affects strategic voting. For low intermediate value, information is predicted to boost strategic voting (behavioral prediction 3). More specifically, MLE predicts the fraction of strategic votes to be 0.06 and 0.16, respectively, for uninformed and informed voters. We observe fractions equal to 0.05 when voters are uninformed and 0.17 when they are informed (*cf*. Table 2). In support of the prediction, the observed increase is statistically significant (*MW*, *Z* = −2.882, *p* < 0.01, *N* = 12). For high intermediate values, we observe on average 0.19 and 0.25 strategic votes for the uninformed and informed cases, respectively, where 0.31 is predicted for both cases. This difference is not statistically significant (*MW*, *Z* = −1.761, *p* = 0.09, *N* = 12).

Finally, note that Fig. 5 shows little evidence of learning across elections. As mentioned above, our design with new preference draws in each election is not favorable for learning (the only trend seems to be a decline in strategic voting in the first 15 elections when subjects are uninformed and the intermediate value is low). It is noticeable that the QRE predictions fare well even in this environment where learning is difficult.

### Voter types

**Fig. 6:**
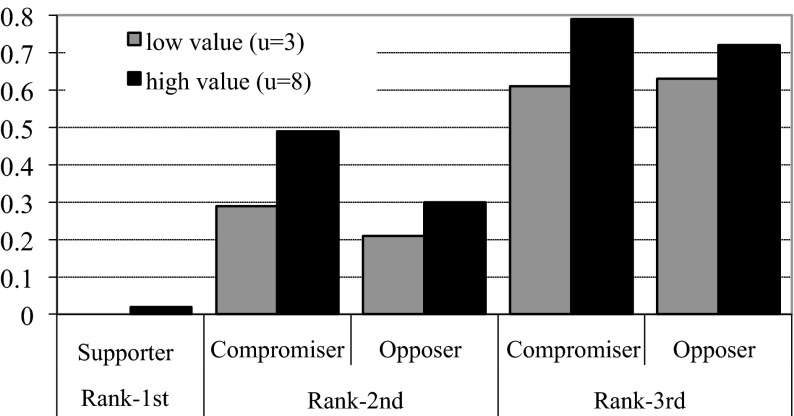
Strategic voting and voter types. *Bars* show for the informed treatment the fraction of votes that were strategic. Voter types are distinguished along the* horizontal axis* and the intermediate value treatments by the *color* of the* bar*

Next, consider the variation of strategic behavior across voter types (Rank-Types and Incentive Types). Figure 6 shows this for the treatment with information (without information, voters do not know their type). We clearly observe that Rank 3rd voters vote more strategically. In fact, they vote more often strategically than sincerely. Rank 1st voters basically never vote strategically and Rank 2nd voters vote strategically often, but less than half of the time.

Behavioral prediction 4 is that Rank 3rd voters vote strategically more often than the other rank types. Tests support this prediction and also show that Rank 2nd types vote strategically more often than supporters do. This holds for both low and high intermediate value (in all comparisons: *W*, *Z* = −2.201, *p* = 0.03; *N* = 6).^12^ For a further comparison of Rank 2nd and Rank 3rd types, we distinguish between opposers and compromisers in both cases. The difference between strategic voting of Rank 3rd and 2nd types is statistically significant for compromisers as well as for opposers. This again holds for low and high intermediate values (all tests, *W*, *Z* = −2.201, *p* = 0.03; *N* = 6). Together, these results provide strong support for the fourth behavioral prediction.

Behavioral prediction 5 is that Opposers vote more strategically than Compromisers for low intermediate value. We observe the opposite: less strategic voting by Opposers (0.29) than by Compromisers (0.44). The difference is statistically significant (*W*, *Z* = −1.992, *p* = 0.05, *N* = 6), a clear rejection of prediction 5. For high intermediate value, behavioral prediction 6 is that Compromisers will vote more strategically than Opposers. This is indeed observed in our data, where the fraction of strategic votes is 0.63 and 0.38, respectively. The difference is statistically significant (*W*, *Z* = −2.201, *p* = 0.03, *N* = 6), in support of the prediction.

All of the previous tests have been univariate and based on average results per electorate. To increase the power of the tests we consider the data as deriving from a panel where every participant votes in 40 elections and conduct a probit regression explaining the individual choice to vote at a particular election, with random effects at the electorate level. Table 3 presents the estimated marginal effects. We have added a variable indicating the period number (divided by 10) to allow for learning effects (which we again do not observe). We also added a dummy variable indicating elections where one of the options was supported by an absolute majority, because in this case strategic voting may simply be seen as futile. This was the case in 27.5 % of the elections. The results show that the probability of voting strategically is 3.5 % points lower in these rounds, when the intermediate value is high (the effect for low value is statistically insignificant). Other factors remain important, however.

**Table 3:** Strategic voting

	Intermediate value	
	Low (*u* ^*m*^ = 3)	High (*u* ^*m*^ = 8)
Constant (coefficient)	−2.677**	−1.947**
Period/10	0.006	0.003
Simple majority	0.013	−0.035*
Compromiser	0.148	0.306**
Opposer	0.079	0.207**
Rank 2nd	0.207**	0.247**
Rank 3rd	0.542**	0.567**
Rank 2nd × opposer	0.002	−0.056
Rank 3rd × opposer	0.059	0.019
Test rank 2nd = Rank 3rd	*χ* ^*2*^ = 53.5 (*p* < 0.001)**	*χ* ^*2*^ = 48.8 (*p* < 0.001)**
Test compromiser = opposer	*χ* ^*2*^ = 0.34 (*p* = 0.559)	*χ* ^*2*^ = 0.74 (*p* = 0.390)
Test rank 2nd: compromiser = opposer	*χ* ^*2*^ = 8.53 (*p* = 0.004)**	*χ* ^*2*^ = 32.9 (*p* < 0.001)**
Test rank 3rd: compromiser = opposer	*χ* ^*2*^ = 0.220 (*p* = 0.641)	*χ* ^*2*^ = 1.78 (*p* = 0.182)

The table presents the results of a random effects probit regression model where the dependent variable is a dummy indicating whether or not voter *i* in electorate j voted strategically in election *t*. Formally, it gives the marginal effects derived from the regression model ${ \Pr }_{t}^{ij} = \varPhi \left( {X_{t}^{ij} \,\,'\beta + \mu^{j} } \right)$ where $\Pr_{t}^{ij}$ gives the probability that *i* of *j* votes strategically in *t*. $\varPhi$ denotes the cumulative normal distribution and *X* is the vector of independent variables described in the first column of the table. *µ* ^*j*^ is a (white noise) electorate-specific error that corrects for the dependencies across individual decision in the same group. The independent variable ‘Simple Majority’ is a dummy variable indicating situations where one of the preference orderings had an absolute majority of at least 7. The independent variables with an ‘x’ between variables indicate interaction terms. To avoid the dummy trap, the variable indicating Rank 1st voters (i.e., Supporters) has been left out of the regression. The tests depicted in the last two rows test equality of the estimated coefficients. Our results are not sensitive to the choice of quadrature points; when varying these points all differences are smaller than 10^−8^

* (**) denotes statistical significance at the 5 % (1 %)-level

The results also show that, irrespective of the intermediate value, Rank 2nd and 3rd voters are both more likely to vote strategically than Supporters (the category absorbed in the constant term), and that Rank 3rd voters are most likely to vote strategically. This confirms the results from our univariate analyses. In fact, Rank 3rd voters have a 54–57 %-points higher probability of voting strategically than Supporters. The effect of incentive-type is smaller. With low intermediate value it does not matter statistically whether one is Supporter, Compromiser, or Opposer. When *u* ^*m*^ = 8, both Compromisers and Opposers vote more often strategically than Supporters do, but the difference between the two is statistically insignificant. Com-promisers have a 31 %-point higher probability of voting strategically than Supporters do.

### Summarizing the results

MLE predicts behavior in the experimental setting reasonably well. Data support various behavioral predictions at the aggregate level as well as the comparative statics for our treatments. In particular, we observe that

- With and without information, the probability of strategic voting is increasing in the importance of the intermediate option;
- With low intermediate value, there is more strategic voting with information than without;
- With high intermediate value there is no statistically significant effect of information.

When considering behavior disaggregated per type of voter, our data support the prediction that

- With full information Rank 3rd voters are more likely to vote strategically than Rank 2nd voters (and both more often vote strategically than Rank 1st do).

and we observe that

- Compromisers vote more strategically than Opposers and both more than Supporters.

MLE predicts this comparative statics only for high intermediate value.

## Concluding remarks

We study a voting environment characterized by the regular occurrence of Condorcet cycles in preferences. Voters are faced with the decision of voting sincerely or strategically. They know their own preference, but may or may not have information about the distribution of preferences across the electorate. When this information is available, certain characteristics of this distribution (such as the rank of the support for one’s most preferred candidate or the relative position of the plurality-preferred candidate in one’s preference ordering) may become important in determining what to vote for. The way such factors affect the probability of voting strategically is partly captured by the predictions derived by adding bounded rationality to a standard utilitarian voting model and deriving the MLE.

Our goal has been to establish whether or not people vote strategically, and what factors affect the probability of doing so. We excluded one obvious candidate for strategic voting, i.e., situations with a Condorcet loser. Instead, we created an environment in which options are a priori symmetric and where Condorcet cycles are likely to occur. In this environment, one that is regularly observed in the field, a strategic vote aims at securing one’s second-preferred option instead of trying to have the most-preferred option win. Our boundedly-rational equilibrium model allows us to derive theoretical predictions on strategic voting. In this way, the equilibrium analysis provides an important tool for understanding the strategic vote. In the end, whether or not voters vote strategically is an empirical question, however. Laboratory control has allowed us to provide answers to this question. We know exactly when a subject in the experiment votes strategically. By varying model parameters one at a time, we were able to establish that voters vote more strategically when the relative value of the second-preferred option increases but that knowing the distribution of preferences makes strategic voting more likely if this relative value is low.

Laboratory control has also allowed us to study in detail *who* votes strategically. We find strong evidence for the (intuitive) MLE prediction that voters who prefer the candidate with the largest support (the ‘Majoritarian Candidate’) vote sincerely for this candidate. For strategic voting by other voters, two characteristics of their preferences may play a role. First, it may matter whether a voter has the Majoritarian Candidate as a second or third preferred option. In the former case, she may decide to vote strategically in an attempt to help the supporters of this candidate to obtain a majority. Second, it may matter how a voter’s most preferred candidate ranks in a poll where everyone votes sincerely. If this rank is lowest, the voter may vote strategically believing that her most preferred candidate does not stand a chance. Our data show that the second argument is more important than the first. Compared to a supporter of the Majoritarian Candidate, a supporter of the lowest ranked candidate has a more than 50 %-point higher probability of voting strategically.

Of course, a downside of using laboratory experiments is that we were forced to restrict the analysis to committee-size voting bodies. The confirmation of the main MLE-predictions for committees does give some confidence in their predictions for larger voting bodies, however (see Levine and Palfrey 2007 for a similar argument with respect to voter turnout experiments). Moreover, there is ample empirical evidence (reviewed in Sect. 1) that substantial strategic voting takes place even at national elections. Hence, the question is not whether strategic voting takes place, but what the causes and effects of strategic voting are. Our design and results pertain to this question.

In our introduction, we argued that a sufficient condition for correct aggregation of preferences is that every voter casts a vote for her most preferred alternative. If this occurs, the winner is in the set of Majoritarian Candidates 100 % of the time. In our experiment, we observe this 72–88 % of the time with uninformed voters and 93–96 % of the time when voters know each others’ preferences (*cf*. Fig. 4). From our laboratory results, we therefore conclude that revelation of the distribution of preferences (e.g., through opinion polls) is sufficient for voting to correctly aggregate preferences in this way.^13^ With such polls, the plurality’s desire is usually honored, even when some voters vote strategically. We conclude that information works as a coordination device around the victory of the Majoritarian Candidate. In summary, information impacts voting behavior by increasing strategic behavior in some situations, differentiating voting patterns across types, and promoting a higher chance of victory for the Majoritarian Candidate.

## Electronic supplementary material

Below is the link to the electronic supplementary material.

Supplementary material 1 (PDF 1334 kb)

## Footnotes

1 For (indirect) empirical evidence of strategic voting in legislatures, see Clinton Joshua and Meirowitz (2004). Using a clever identification strategy, Kawai and Watanabe (2013) estimate that only a small fraction of Japanese voters engage in strategic voting (which they call ‘misaligned’ voting) in Japanese House of Representatives elections. For large electorates Cain (1978) and Myatt and Fisher (2002) provide empirical evidence from the UK, Alvarez and Roderick Kiewiet (2009) from California, and Blais and Nadeau (1996) and Blais et al. (2001) from Canada. A difficulty in using field data to empirically assess the occurrence of strategic voting is the fact that it requires knowledge of the voters’ preference orderings over the alternatives. Some have tried to overcome this by eliciting preferences using survey questions (Cain 1978; Blais and Nadeau 1996; Blais et al. 2001; Myatt and Fisher 2002). Such measurement is subject to noise and strategic reporting, however. Moreover, while analyses using observational field data allow one to study the occurrence of strategic voting per se, they do not really allow for a systematic study of its causes and consequences.

2 Duverger’s law predicts that in a plurality vote the votes will converge to two candidates, mainly due to the psychological phenomenon of the ‘wasted vote’. Voters supporting a candidate with low perceived chances of winning are assumed likely to move their vote to the more preferred option of the leading two (e.g., Riker 1982b; Palfrey 1989).

3 Alternatively, one could consider sequential elections like the U.S. primaries, where information about others’ preferences may be obtained from previous elections. Morton and Williams (1999, 2001) use this to study information asymmetries regarding candidates’ identities. The information we provide can be seen as a ‘noiseless’ early opinion poll, reflecting more the actual preferences than the intended voting behavior. Early polls are not necessarily good predictors of election outcomes. Opinion polls closer to the election tend to capture more strategic behavior and aim basically at predicting the election outcome (McKelvey and Ordeshook 1986; Gelman and King 1993; Brown et al. 1999; Erikson and Wlezien 2001).

4 Our results are robust to heterogeneity in *u* ^*m*^ (Tyszler and Schram 2013).

5 There are also equilibria in which members of the same group act differently. Take *K* even and *K* ≥ 6. Assume two groups voting sincerely. If the remaining group has half of its members voting sincerely and half voting for their second most preferred alternative, the voting outcome could be *v* = (*K*, *K*/2, 3 *K*/2). Once again nobody is pivotal and this is an equilibrium.

6 Voting sincerely is strictly profitable if (*u* ^*b*^ *+* *u* ^*m*^ *+* *u* ^*l*^)/3 > *u* ^*m*^ ⟺ *u* ^*m*^ < 5.5, where we use *u* ^*b*^ *=* 10 and *u* ^*l*^ = 1. Therefore if the value attributed to the intermediate option is low enough, voting sincerely and creating a tie is the best response. If it is high enough, voting strategically is the best response.

7 For example. Goeree and Holt (2005) use data on the participation games reported by Schram and Sonnemans (1996a, b) and estimate 0.8 for early rounds and 0.4 for late rounds.

8 We deal with ties as follows. If (*N*_*ABC*_ *, N*_*BCA*_ *, N*_*CAB*_) = (4, 4, 4), all voters are ranked 1st. If the Majoritarian Set has two elements, supporters of the Majoritarian Candidate are ranked 1st, supporters of the other candidate in the set are ranked 2nd and the remaining voters are ranked 3rd. If the Majoritarian Set is singleton and the two other preference orderings have equal support, all voters not supporting the Majoritarian Candidate are ranked 2nd.

9 Two differences with the experiments described here are an electorate size of 15 voters and a within-subjects variation in information. Two electorates participated in the pilot.

10 Strategic behavior is not concentrated in a few subjects. A between subjects heterogeneity analysis shows very low variability (the standard deviation is smaller than 0.03), indicating that aggregate average behavior is a good indicator of individual behavior.

11 In case of ties (i.e., randomly chosen winner), we did as follows. If only one of the tied options was in the Majoritarian Set, we counted this as a 0.5 success. If one of the two (three) winners was the Majoritarian Candidate we counted the winner as 0.5 (0.33).

12 The reason why the test statistic often has the same value is that in all tests, the ranks are unanimous across the 6 electorates for any comparison. 6 out of 6 positive ranks gives *Z* = 2.201 and *p* = 0.028 in the Wilcoxon test.

13 Of course, this may create an incentive to strategically misreport preferences in a poll. This can easily be studied in future experiments.

## References

[CR1] Alvarez R Michael, Roderick Kiewiet D (2009). Rationality and rationalistic choice in the california recall. British Journal of Political Science.

[CR2] Austen-Smith D, Jeffrey Banks S (1996). Information aggregation, rationality, and the Condorcet jury theorem. American Political Science Review.

[CR3] Blais A, Nadeau R (1996). Measuring strategic voting: a two-step procedure. Electoral Studies.

[CR4] Blais A, Nadeau R, Gidengil E, Nevitte N (2001). Measuring strategic voting in multiparty plurality elections. Electoral Studies.

[CR5] Brown LB, Chappel HW (1999). Forecasting presidential elections using history and polls. International Journal of Forecasting.

[CR6] Cain BE (1978). Strategic voting in Britain. American Journal of Political Science.

[CR7] Clinton Joshua D, Meirowitz A (2004). Testing explanations of strategic voting in legislatures: A reexamination of the compromise of 1790. American Journal of Political Science.

[CR8] Cox G (1997). Making votes count.

[CR9] Downs A (1957). An economic theory of political action in a democracy. The Journal of Political Economy.

[CR10] Erikson RS, Wlezien C (2001). Presidential polls as a times series: The case of 1996. Public Opinion Quarterly.

[CR11] Farquharson R (1969). Theory of voting.

[CR12] Fey M (1997). Stability and coordination in Duverger’s law: A formal model of preelection polls and strategic voting. American Political Science Review.

[CR13] Fischbacher U (2007). Z-tree: Zurich toolbox for ready-made economic experiments. Experimental Economics.

[CR14] Forsythe R, Myerson R, Rietz T, Weber R (1993). An experiment on coordination in multi-candidate elections: The importance of polls and election histories. Social Choice and Welfare.

[CR15] Forsythe R, Rietz T, Myerson R, Weber R (1996). An experimental study of voting rules and polls in three-candidate elections. International Journal of Game Theory.

[CR16] Gelman A, King G (1993). Why are American presidential election campaign polls so variable when votes are so predictable. Britsh Journal of Political Science.

[CR17] Gerber E, Morton RB, Rietz T (1988). Minority representation in multimember districts. American Political Science Review.

[CR18] Goeree JK, Holt CA (2005). An explanation of anomalous behavior in models of political participation. American Political Science Review.

[CR19] Goldfinger, J. (2004). The normative implications of cyclical majorities in theory and practice. In *Workshop on Political Theory and Public Policy*.

[CR20] Gratschew, M. (2001). Compulsory voting. IDEA - International Institute for Democracy and Electoral Assistance, from http://www.idea.int/vt/compulsoryvoting.cmf.

[CR21] Groβer J, Schram A (2010). Public opinions polls, voter turnout, and welfare: An experimental study. American Journal of Political Science.

[CR22] Kawai K, Watanabe Y (2013). Inferring strategic voting. American Economic Review.

[CR23] Lehtinen, A. (2007). The welfare consequences of strategic voting. *PhD*-*thesis* EUR.

[CR24] Levine DK, Palfrey T (2007). The paradox of voter participation? A laboratory study. American Political Science Review.

[CR25] McKelvey RD, Ordeshook PC (1986). Information, electoral equilibria and the democractic ideal. The Journal of Politics.

[CR26] McKelvey RD, Palfrey T (1995). Quantal response equilibria for normal form games. Games and Economic Behavior.

[CR27] Morton RB, Williams KC (1999). Information asymmetries and simultaneous versus sequential voting. American Political Science Review.

[CR28] Morton RB, Williams KC (2001). Learning by voting.

[CR29] Myatt DP, Fisher SD (2002). Tactical coordination in plurality electoral systems. Oxford Review of Economic Policy.

[CR30] Nitzan S, Procaccia U (1986). Optimal voting procedures for profit maximizing firms. Public Choice.

[CR31] Palfrey T, Models of Strategic Choice in Politics (1989). A mathematical proof of Duverger’s Law. Peter ordershook.

[CR32] Palfrey T, Weingast B, Wittman D (2006). Laboratory experiments in political economy. Handbook of political economy.

[CR33] Riker WH (1982). Liberalism against populism.

[CR34] Riker WH (1982). The two-party system and Duverger’s law: An essay on the history of political science. American Political Science Review.

[CR35] Schram A, Sonnemans J (1996). Voter turnout as a participation game: An experimental investigation. International Journal of Game Theory.

[CR36] Schram A, Sonnemans J (1996). Why people vote: Experimental evidence. Journal of Economic Psychology.

[CR39] Tyszler M, Schram A (2013). Voting in heterogeneous electorates: An experimental study. Games.

