# Supplementary material for: Information and strategic voting
Source: Exp Econ. 2015 May 17;19(2):360–81. doi: 10.1007/s10683-015-9443-2 (PMC5153667; doi:10.1007/s10683-015-9443-2)
Supplement: Supplementary file 1 — Supplementary material 1 (PDF 1334 kb) [file 10683_2015_9443_MOESM1_ESM.pdf]

Online Appendix

Appendix A: Quantal Response Equilibrium

Appendix B: Voting probabilities in the MLE

Appendix C: Principal branch of the MLC

Appendix D: Limiting MLE

Appendix E: MLC for large electorates

Appendix F: MLC per voter type

Appendix G: Experimental instructions

Appendix H: Preference distributions used

Appendix A - Quantal Response Equilibrium

In this appendix, we describe how one can derive the QRE for our voting game. To find the QRE, we first consider the expected utility derived from voting for distinct options. Take, for example, voter *i* with preference ordering (*A,B,C*). The expected payoff from voting for *A*, u_A^e, depends on what other voters do. It is a function of the probabilities with which other voters vote for the three options. Similarly, the expected utility from voting for *B* and *C*, u_B^e and u_C^e, depend on these probabilities. Nash equilibrium analysis assumes that *i* will vote for the alternative that gives her the highest expected utility, *i.e.*, she gives the best response to others' probabilities.

In contrast, a QRE analysis allows *i* to make errors in the vote decision. One way to do this is by adding a stochastic term to the expected utility functions, yielding expected utilities $u_A^e + \mu\varepsilon_A$, $u_B^e + \mu\varepsilon_B$, and $u_C^e + \mu\varepsilon_C$ for *A*, *B*, and *C*, respectively. In these terms, $\mu \geq 0$ is an error parameter and the ε terms are i.i.d. realizations of random variables. This parameterization is general enough to capture different sources of noise, such as distractions, perception biases, miscalculations or limited computational capability (Goeree and Holt, 2005).

A voter will still vote for the option with the highest expected utility but this is now a stochastic event. For example, she will vote for *A* if $u_A^e + \mu\varepsilon_A > u_B^e + \mu\varepsilon_B$ and $u_A^e + \mu\varepsilon_A > u_C^e + \mu\varepsilon_C$ or

$$\varepsilon_B - \varepsilon_A < \frac{u_A^e - u_B^e}{\mu} \text{ and } \varepsilon_C - \varepsilon_A < \frac{u_A^e - u_C^e}{\mu} \qquad \text{(A1)}$$

Specification of the distribution functions of ε_A, ε_B, ε_C yields the probability that *i* will vote for option *A* (and similarly for *B* or *C*). Assuming that the ε's follow the extreme value type 1 distribution, the (multinomial) probability that *i* will vote for option j, p_j^i, is given by:

$$p_j^i = \frac{\exp[u_j^e / \mu]}{\sum_{l=A,B,C} \exp[u_l^e / \mu]}, \quad j = A, B, C. \qquad \text{(A2)}$$

Next, recall that the probabilities of other voters choosing *A*, *B*, or *C* enter the expected utility terms in the right hand side (r.h.s.) of (A2). A full specification for all voters then equates a vector of (3N) voting probabilities on the left hand side (l.h.s.) to a vector of functions of the same probabilities on the r.h.s.. A QRE (specifically, a 'multinomial' logit equilibrium, MLE) is defined as a vector of probabilities that when entered on the r.h.s. yields itself on the l.h.s.

Here, the MLE depends on μ, u^m, N_{ABC}, N_{BCA}, *and* N_{CAB}, and on whether or not voters know the latter three numbers. To understand the role of the error parameter μ, note that

$$\lim_{\mu \downarrow 0} p_j^i = \begin{cases} 0, & if\ u_j^e < \max_k \{u_k^e\} \\ 1, & if\ u_j^e = \max_k \{u_k^e\}\ and\ u_l^e < u_j^e, l \neq j \end{cases} \tag{A3}$$

(and $\lim_{\mu \downarrow 0} p_j^i$ is 1/K if K options (K=2,3) yield equal maximum expected utility).

It follows directly from (A3) that as noise diminishes to zero, the option with the highest expected utility is chosen, i.e., the MLE converges to a Nash equilibrium (see McKelvey and Palfrey 1995). Similarly,

$$\lim_{\mu \to \infty} p_j^i = \frac{1}{3},\ j = A, B, C, \tag{A4}$$

which shows that behavior converges to pure randomization as noise increases to infinity.

One can compute MLE for any positive and finite value of μ. We call the set of MLE and correspondent μ's the 'Multinomial Logit Correspondence' (MLC). Except for the limit case where μ approaches infinity, there need not be a unique MLE. One can, however, identify a unique branch of the MLC that starts from the limit at $\mu = \infty$ and continuously converges to a unique Nash Equilibrium as $\mu \downarrow 0$ (McKelvey and Palfrey, 1995). This is called the 'Principal Branch' and the corresponding Nash Equilibrium the 'limiting MLE' of the game.[1] In this way, one can use the principal branch of the MLC as a selection device for the set of Nash equilibria. Though other equilibria may also be limits of the MLE correspondence, the selection by the principal branch is generally unique.[2]

Using the Quantal Response model with the multinomial logit specification has several advantages: (i) it provides a refinement selecting precisely one of the multiple Nash equilibria (i.e., the limiting MLE); (ii) it takes bounded rationality seriously by introducing noise in the individual choice problem; (iii) the principal branch has the intuitive characteristic that players of the same type play symmetric strategies; (iv) in line with intuition, for all finite μ the MLE probability of choosing an option is increasing in the expected payoff differences with other options. The expected payoff difference will vary with the extent of information

[1] Except for very special cases, the principal branch needs to be computed numerically. In order to trace it we use the Homotopy Approach as outlined by Turocy (2005, 2010).

[2] The few cases where the principal branch is not unique are due to backward bending portions of the branch. For these, we select equilibria based on a 'first-pass' rule. In the "first-pass" criteria we select the first equilibrium computed on any given μ when tracing the correspondence from μ = ∞ to μ = 0. The intuitive reasoning is that if any learning process applies, it is more reasonable to assume that it moves from more to less noisy behavior then the other way around. For more details see appendix C.

and the realized distribution[3] but it only includes situations where the voter's choice makes a difference, since for every non-pivotal situation the payoff difference will be 0.

This last point can be illustrated with an example. As can be easily seen, the r.h.s. of (A2) can be rewritten in terms of expected payoff differences, taking voting sincerely as the reference strategy. For example, for a voter with preference ordering (A,B,C), we write:

$$p_A^i = \frac{1}{1+\exp\left[\left(u_B^e - u_A^e\right)/\mu\right] + \exp\left[\left(u_C^e - u_A^e\right)/\mu\right]}$$
$$p_B^i = \frac{\exp\left[\left(u_B^e - u_A^e\right)/\mu\right]}{1+\exp\left[\left(u_B^e - u_A^e\right)/\mu\right] + \exp\left[\left(u_C^e - u_A^e\right)/\mu\right]} \quad \text{(A5)}$$
$$p_C^i = \frac{\exp\left[\left(u_C^e - u_A^e\right)/\mu\right]}{1+\exp\left[\left(u_B^e - u_A^e\right)/\mu\right] + \exp\left[\left(u_C^e - u_A^e\right)/\mu\right]} \quad .$$

The expected utility difference of voting for option *j* instead of *k* $u_j^e - u_k^e$, is a weighted sum of the utility differences between voting for *j* or *k* for all possible combinations of votes by other voters (denote by –*i*): $u_j^e - u_k^e = \sum_{-i} P_{-i}(u_j^{-i} - u_k^{-i})$, where P_{-i} denotes the probability that a particular configuration of other voters' choices occurs and u_j^{-i} (u_k^{-i}) gives the expected utility obtained from choosing *j* (*k*) in situation –*i*. Though there are an extreme number of situations –*i*, for most of them *i*'s vote will not affect the outcome. In those situations, $u_j^{-i} = u_k^{-i}$ so they do not add to the expected utility difference. Therefore, in (A5) the voter takes into account only the relevant pivotal situations. An important consequence is that the probabilities in (A5) converge to 1/3 as the electorate becomes infinitely large. The intuition is that for infinitely large electorates it no longer matters what any single voter does, and random noise dominates the voter's choice. We will further discuss this, below. For details see appendix C.

[3] See appendix B for details of the computations.

Appendix B - Voting probabilities in the MLE

In this appendix we show how the probabilities of voting for various options depends on the probabilities of being pivotal in various situations and how this yields the conclusion that these probabilities converge to 1/3 as the size of the electorate increases to infinity.

The (multinomial) probability that a voter *i* with preference ordering (A,B,C) will vote for option *j=A,B,C,,* is denoted by p_j^i, and given by (A5), which we summarize by

$$p_j^i = \frac{\exp\left[\left(u_j^e - u_A^e\right)/\mu\right]}{1+\exp\left[\left(u_B^e - u_A^e\right)/\mu\right]+\exp\left[\left(u_C^e - u_A^e\right)/\mu\right]} \quad , \; j=A,B,C \qquad \text{(B1)}$$

Recall that the expected utility difference of voting for *j* instead of *k,* $u_j^e - u_k^e$ is a weighted sum of the utility differences between voting for *j* or *k* for all possible combinations of votes by other voters (−*i*). For example:

$$u_A^e - u_B^e = \sum_{-i} P_{-i}(u_A^{-i} - u_B^{-i}) \qquad \text{(B2)}$$

where P_{-i} denotes the probability that a particular configuration of other voters' choices occurs and u_j^{-i} (u_k^{-i}) gives the utility obtained from choosing *j* (*k*). A configuration of other voters' choices depends on the configuration of their preferences and on their choices conditional on their preferences.

There are $\binom{N+1}{2}$ possible preference configurations for other voters. Each will take the form ($N-1_{ABC}$, $N-1_{BCA}$, $N-1_{CAB}$), and will occur with (multinomial) probability:

$$P_{(N-1_{A,B,C},N-1_{B,C,A},N-1_{C,A,B})} = \frac{(N-1)!}{\left(N-1_{A,B,C}\right)!\left(N-1_{B,C,A}\right)!\left(N-1_{C,A,B}\right)!}\left(\frac{1}{3}\right)^{(N-1)} \qquad \text{(B3)}$$

In each of these, the probabilities of various configurations of the others' votes depends on their strategies, *i.e.*, the probabilities with which they vote for *A, B,* or *C.* These then determine the probabilities that *i* will be pivotal. For all non-pivotal situations, $u_A^{-i} = u_B^{-i} = u_C^{-i}$.

It follows directly from (B2) that only pivotal probabilities are relevant in determining the expected utility differences in (B1).

To illustrate, consider the configuration of other voters preferences (*N-1,0,0*), *i.e,* all other voters have preference ordering (*A,B,C*), which occurs with probability $P_{(N-1,0,0)}=(1/3)^{N-1}$. For simplicity, consider only quasi-symmetric strategies.[1] One of the pivotal situations faced by a voter with preference (*A,B,C*) is a tie between *A* and *B*. This occurs with probability:

$$P_{A=B|(N-1,0,0)} = \sum_{i=\left\lceil \frac{N-1}{3} \right\rceil}^{\left\lfloor \frac{N-1}{2} \right\rfloor} \frac{(N-1)!}{i!i!(N-1-2i)!} \left(p_A^{(A,B,C)}\right)^i \left(p_B^{(A,B,C)}\right)^i \left(p_C^{(A,B,C)}\right)^{(N-1-2i)} \qquad \text{(B4)}$$

where $P_{A=B|(N-1,0,0)}$ denotes the probability that a tie occurs between A and B conditional on the distribution of others' preferences being (*N−1*,0,0), and $\lceil x \rceil$ ($\lfloor x \rfloor$) indicates the rounding up (down) of *x*. Note that the sum is restrained to consider only situations where *C* receives fewer votes than *A* and *B* or a three-way tie, i.e., a vote for *A* is decisive in favor of *A* while a vote for *B* is decisive in favor of *B*.

One can derive pivotal probabilities as in (B4) for all configurations of voter preferences and strategies and substitute them for P_{-i} in (B2) (whilst neglecting all P_{-i} for non-pivotal situations). Note that as *N* increases each pivotal probability as in (B4) converges to 0. As a consequence, the difference in expected utility in (B2) converges to 0 and the probability of voting for any specific option in (B1) converges to 1/3.

[1] Quasi-symmetric strategies are strategies that are equal for all players with the same preferences and information and facing the same environment.

Appendix C - Principal branch of the MLC

In this appendix we present graphs of the Principal Branch of the Multinomial Logit Correspondence (MLC) for $N = 12$ and $\mu \in [0, 10]$. We present all the 31 unique distributions regarding quasi-symmetric strategies[1]. In some cases, the principal branch contains “backward bending” portions, i.e., the branch does not always moves monotonically w.r.t. to μ. This leads to multiple equilibria. In order to select one of the equilibria in these cases[2] we applied a “first-pass criteria”.[3] In the “first-pass” criteria we select the first equilibrium computed on any given μ when tracing the correspondence from $\mu = \infty$ toward $\mu = 0$. The intuitive reasoning is that if any learning process applies, it is more reasonable to assume that it moves from more to less noisy behavior then the other way around.

The graphs also show average behavior per experimental electorate, plotted over $\mu = 0.55$, the value used for deriving predictions.

Figure C.1: Principal Branch of the MLC

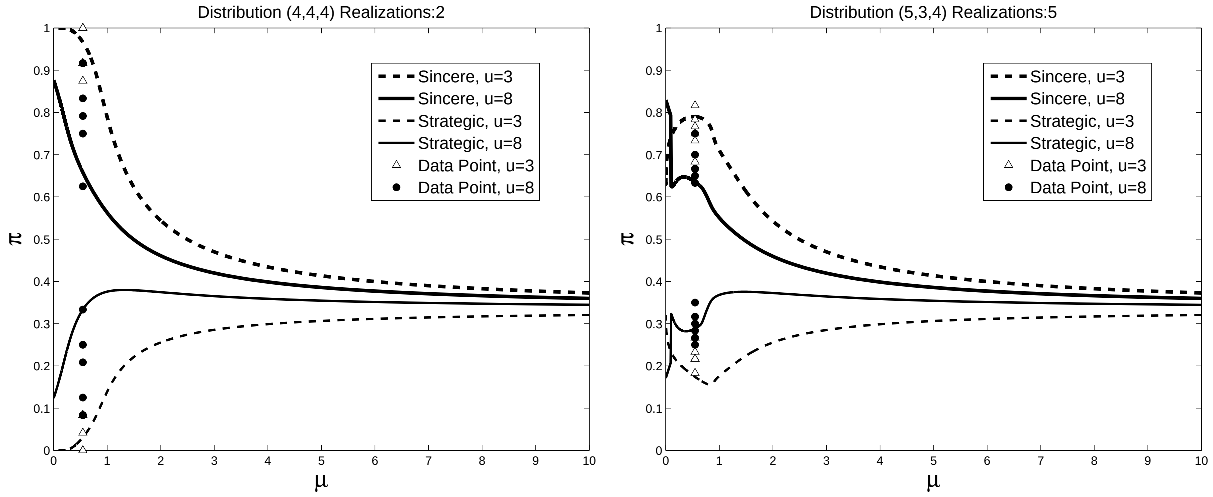

[1]Consider the distributions: $(5, 4, 3)$ and $(3, 4, 5)$. In both cases, the players from the group with 5 voters have as second most preferred option the most preferred option of players from the group with 4 voters. Similarly these voters have as their second most preferred option the most preferred option of players from the group with 3 voters, who, in turn, have as their second most preferred option the most preferred option of the players from the group with 5 voters. Therefore, both distribution have identical MLC when comparing groups by size.

[2]Selection of one equilibrium per distribution is necessary for weighted average computations.

[3]Full graphs are available upon request.

Figure C.1: Principal Branch of the MLC (cont.)

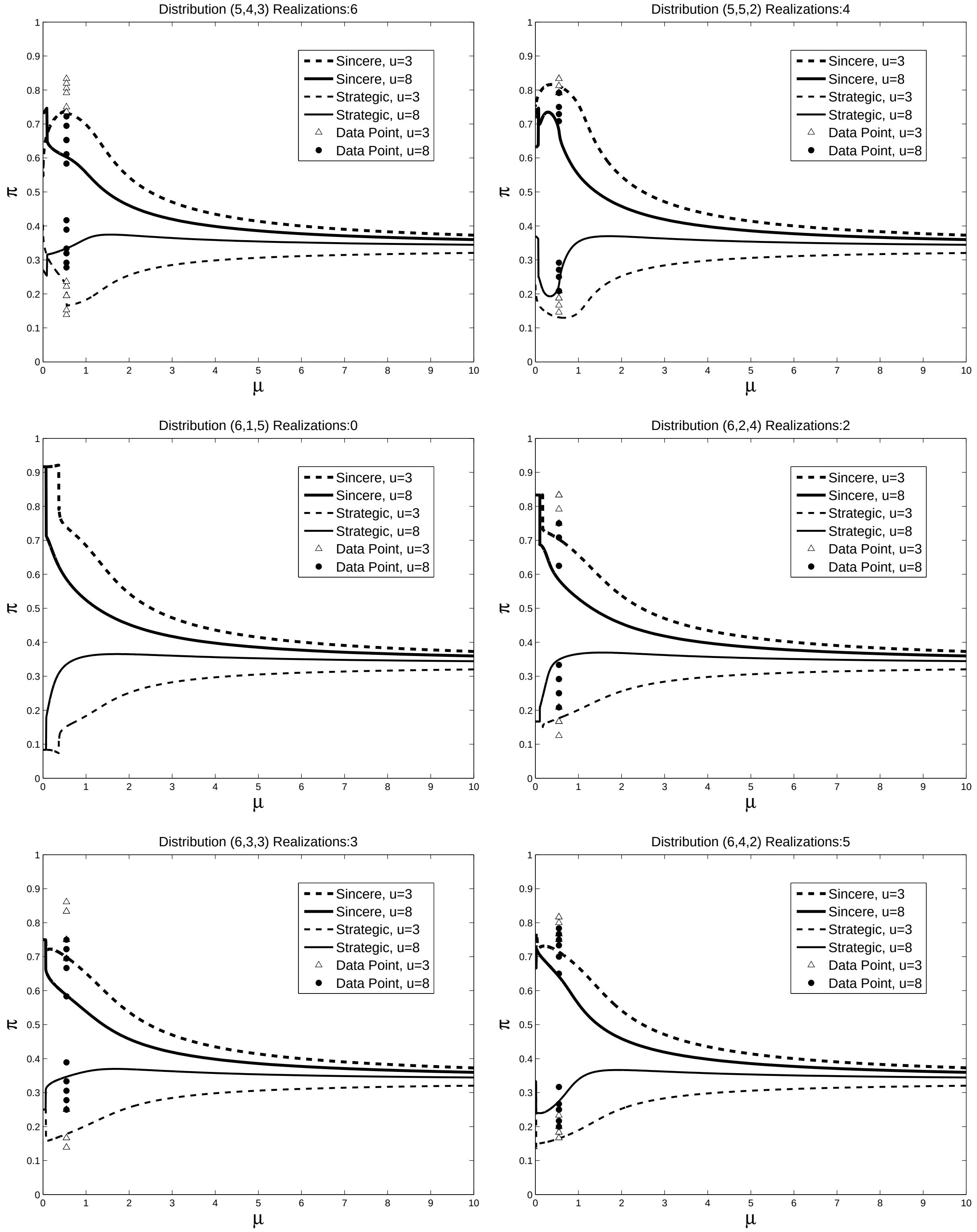

Figure C.1: Principal Branch of the MLC (cont.)

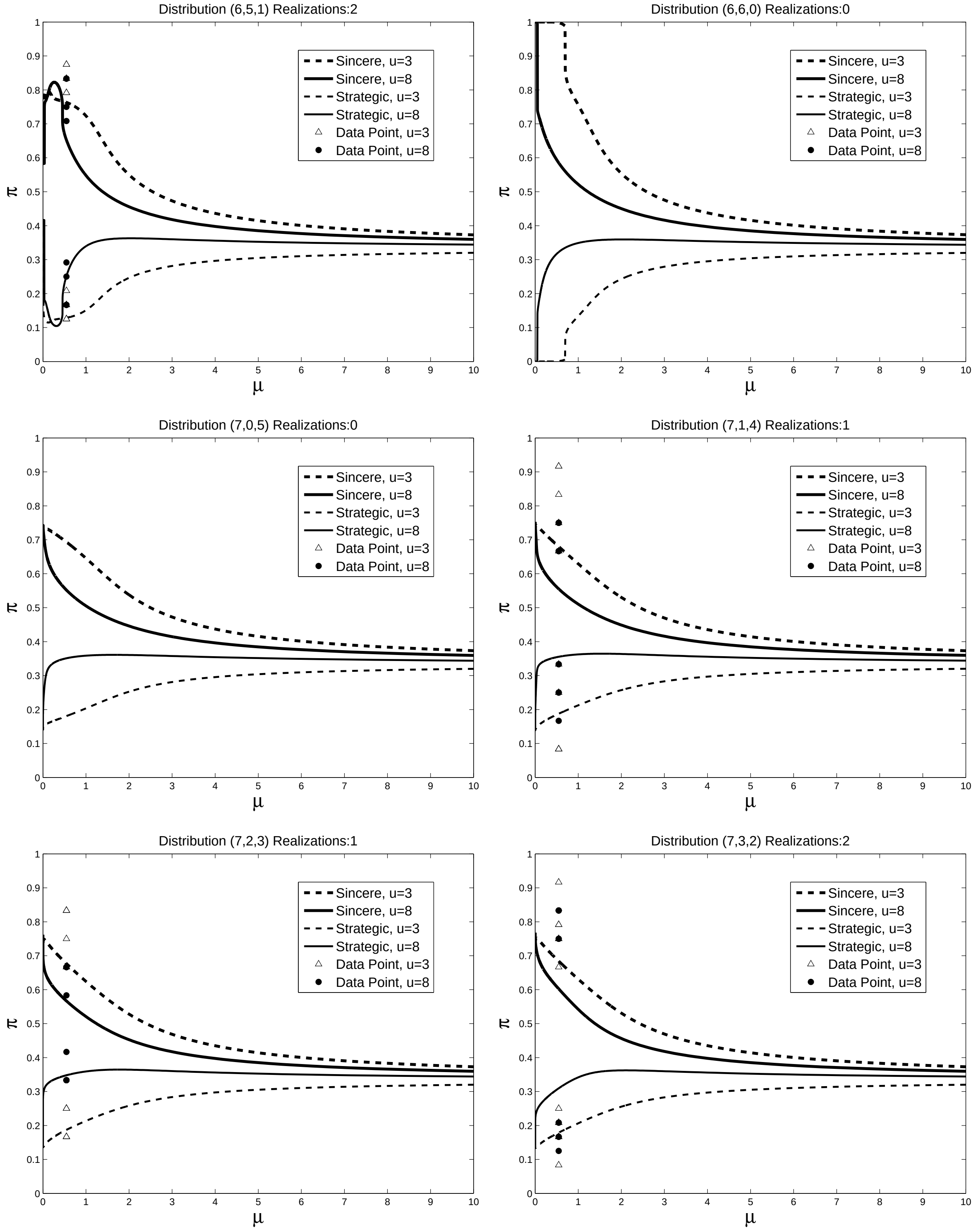

Figure C.1: Principal Branch of the MLC (cont.)

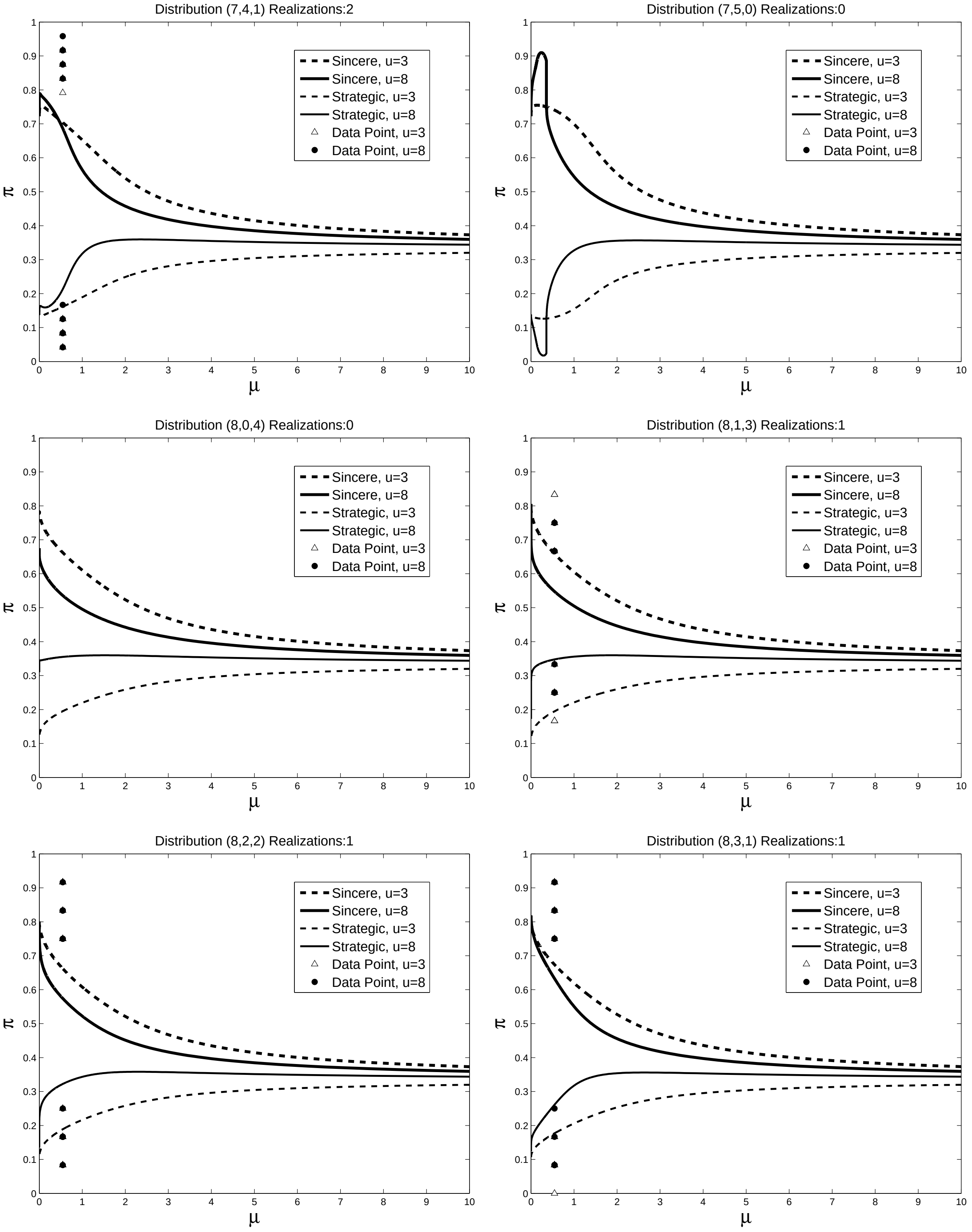

Figure C.1: Principal Branch of the MLC (cont.)

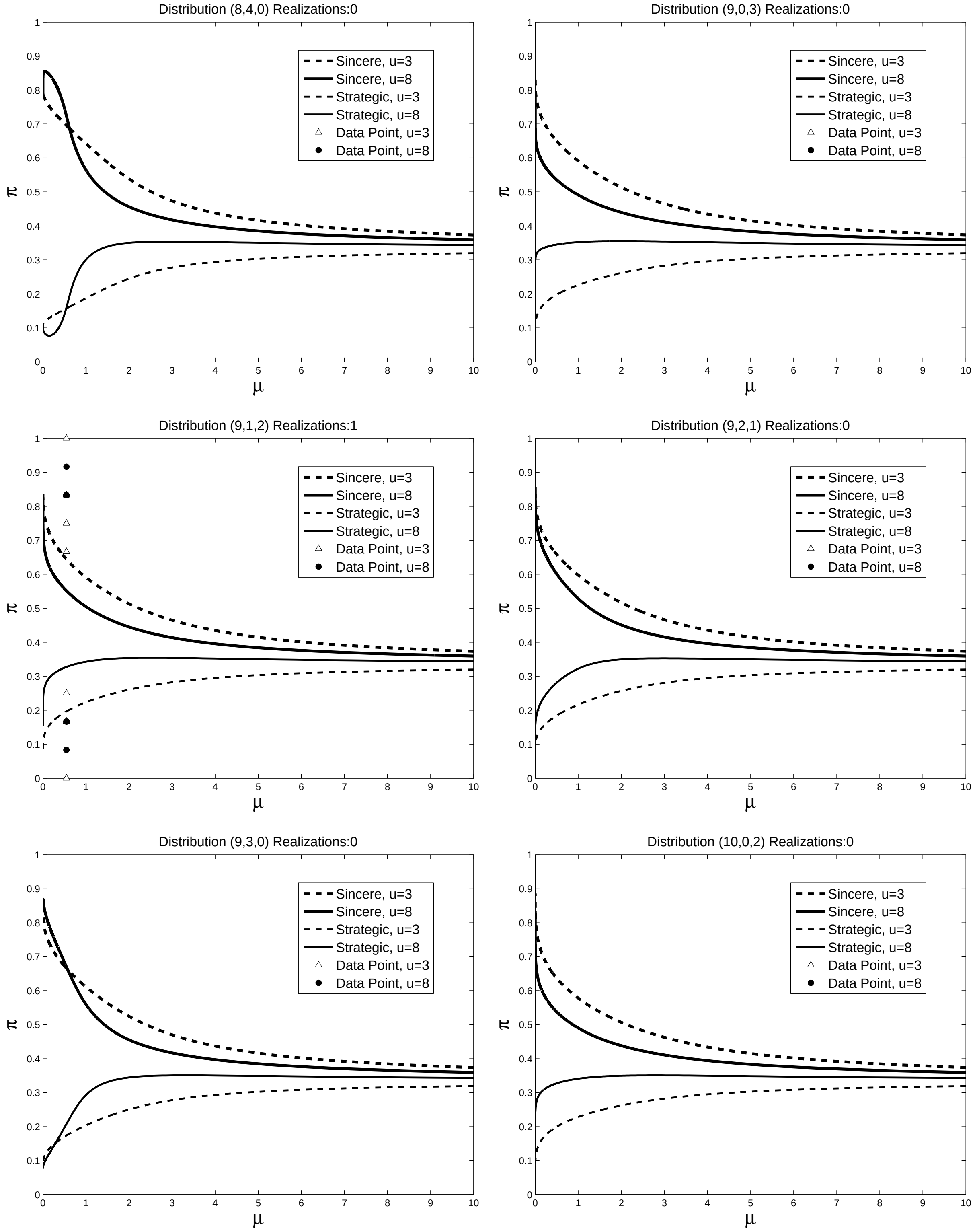

Figure C.1: Principal Branch of the MLC (cont.)

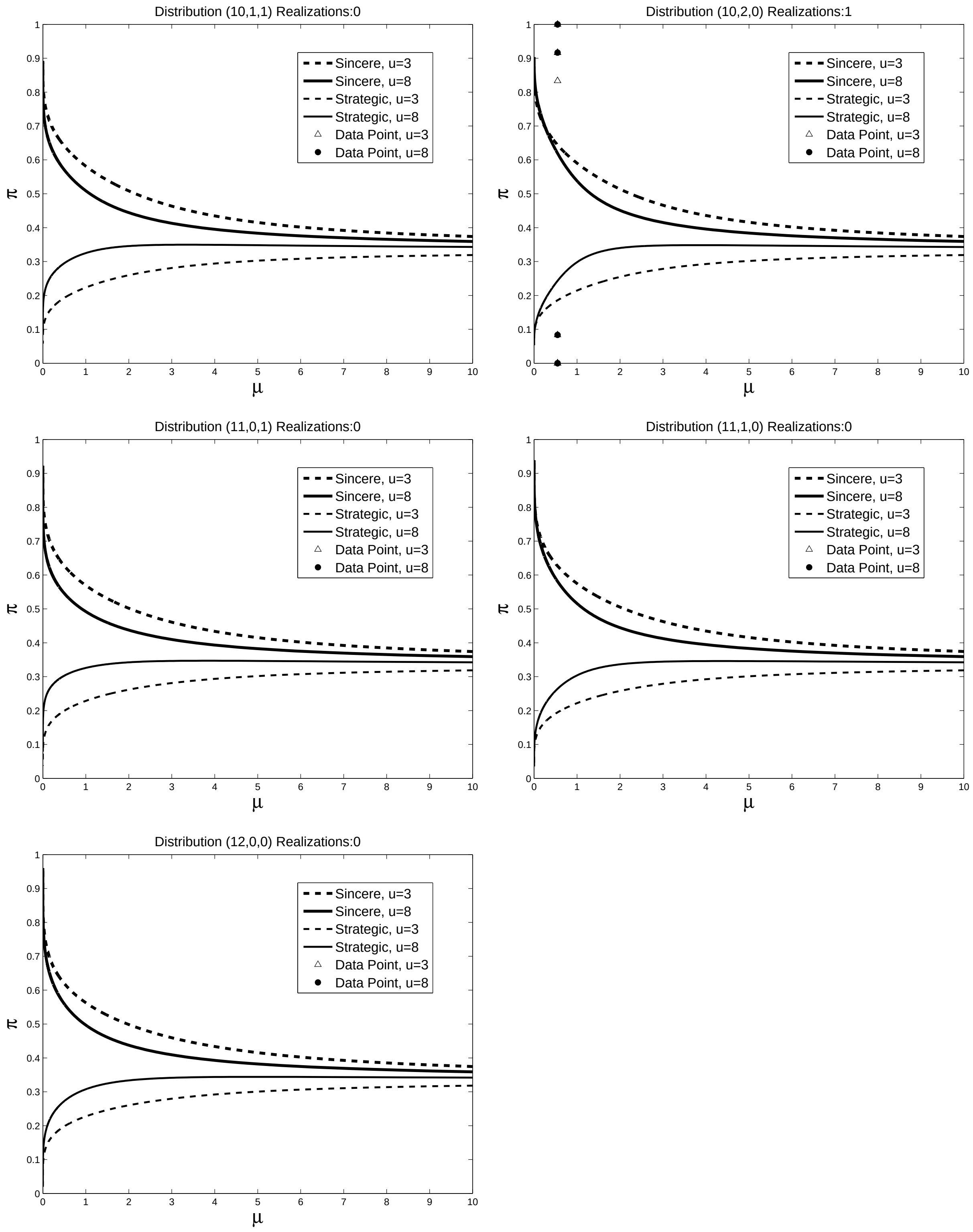

Appendix D - Limiting MLE

This appendix presents the limiting MLE ($\mu = 10^{-6}$) for each unique situation for the informed setting (cf. fn 1 of appendix C).

Table C.1: Limiting MLC, $N = 12$

			Sincere		Strategic		Third Option	
Group 1	Group 2	Group 3	$u^m = 3$	$u^m = 8$	$u^m = 3$	$u^m = 8$	$u^m = 3$	$u^m = 8$
4	4	4	1	0.8767	0	0.1233	0	0
5	3	4	0.671	0.8295	0.3158	0.1705	0.0132	0
5	4	3	0.5865	0.7289	0.3994	0.2711	0.0141	0
5	5	2	0.7572	0.6293	0.2316	0.3707	0.0111	0
6	1	5	0.9167	0.9167	0.0833	0.0833	0	0
6	2	4	0.8333	0.8333	0.1667	0.1667	0	0
6	3	3	0.75	0.75	0.25	0.25	0	0
6	4	2	0.6667	0.6667	0.3333	0.3333	0	0
6	5	1	0.5833	0.5833	0.4167	0.4167	0	0
6	6	0	1	1	0	0	0	0
7	0	5	0.7222	0.7222	0.1389	0.1389	0.1389	0.1389
7	1	4	0.7222	0.7222	0.1389	0.1389	0.1389	0.1389
7	2	3	0.7222	0.7222	0.1389	0.1389	0.1389	0.1389
7	3	2	0.7222	0.7222	0.1389	0.1389	0.1389	0.1389
7	4	1	0.7222	0.7222	0.1389	0.1389	0.1389	0.1389
7	5	0	0.7222	0.7222	0.1389	0.1389	0.1389	0.1389
8	0	4	0.778	0.6708	0.1111	0.3098	0.1109	0
8	1	3	0.778	0.8058	0.1111	0.1724	0.1109	0.0218
8	2	2	0.778	0.7807	0.111	0.1351	0.1109	0.0506
8	3	1	0.7781	0.801	0.111	0.1241	0.1109	0.075
8	4	0	0.7781	0.781	0.111	0.1097	0.1109	0.1093
9	0	3	0.8348	0.7907	0.0836	0.2087	0.0816	0.0007
9	1	2	0.8353	0.8266	0.0832	0.1537	0.0815	0.0197
9	2	1	0.8357	0.8555	0.0829	0.1019	0.0814	0.0426
9	3	0	0.8359	0.8497	0.0827	0.0778	0.0813	0.0725
10	0	2	0.8861	0.8349	0.0587	0.1602	0.0551	0.0049
10	1	1	0.8871	0.8922	0.0579	0.087	0.055	0.0208
10	2	0	0.8877	0.9031	0.0574	0.053	0.0549	0.044
11	0	1	0.9293	0.9127	0.037	0.0788	0.0338	0.0085
11	1	0	0.9305	0.939	0.0359	0.0348	0.0337	0.0262
12	0	0	0.9651	0.9605	0.0179	0.023	0.017	0.0165

Notes. This table shows for each possible unique realization of the preference distribution the average probability of voting sincerely, strategically or for the third option, conditional on the value of the intermediate option. These values are computed using a tracing procedure (Turocy 2005,2010) and reporting the outcome when $\mu = 10^{-6}$.

Appendix E - MLC for large electorates

Here, we describe the MLC for larger N (N=99 and N=999,999). Figure E1 shows the cases for uninformed voters.

Figure E1: Multinomial Logit Correspondences for Uninformed Voters

A

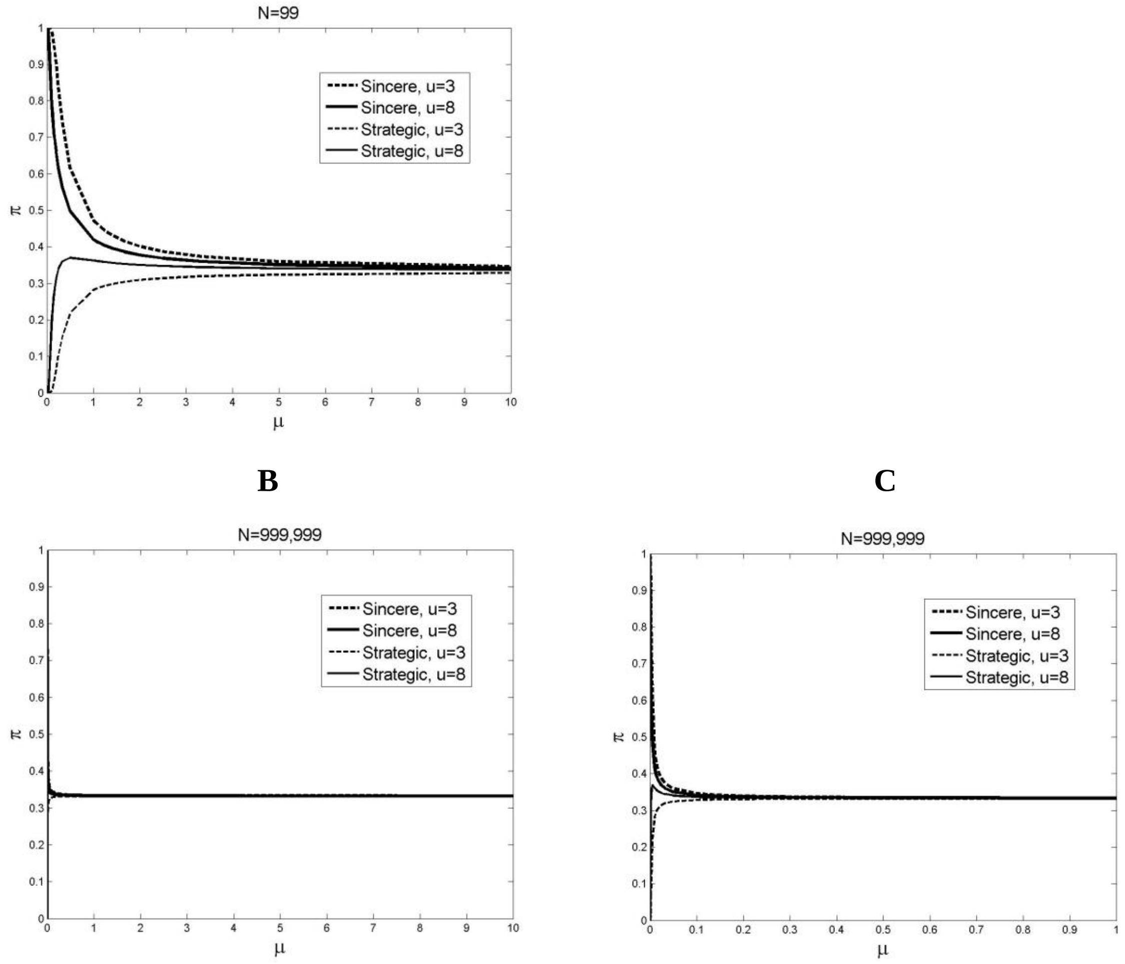

Notes. Lines show the principal branch of the MLC for high (u^m=8) and low (u^m=3) values of the intermediate option. In panels A and B, the size of the voting body (*N*) is 99, and 999,999, respectively. Panel C zooms in on the large electorate case for $\mu \in [0,1]$.

For legislature-size voting bodies (panel A) similar results are obtained as in the N=12 case reported in the main text, though the MLE probability of choosing the dominated alternative increases to approximately 0.2 for μ=1. The probability of voting strategically again depends strongly on the intermediate utility. For $\mu \in (0.4,0.8)$ it is more or less stable around 0.36 when

u^m=8 and increases from ±0.19 to 0.28 for u^m=3. Hence, MLE predicts substantial strategic voting, even in legislature-size groups.

Finally, panels B and C of figure E1 show the MLCs in large electorates (approximately 1 million voters). Here, the probability of being pivotal is so small that the noise term dominates voters' decisions. Even for low μ-values the probability of voting for any of the options is close to 1/3. Only for μ<0.1 can we distinguish between probabilities for the distinct options. Of course, one could easily adapt the model and arrive at non-random equilibrium probabilities of sincere voting.[1] Here we conclude that in large electorates significant effects of our model parameters on the probability of strategic voting are only observed for very low levels of noise.

Figure E2 shows the MLC for informed voters in the case where (N_{ABC},N_{BCA},N_{CAB}) = (33,33,33).

Figure E2: Multinomial Logit Correspondences for Informed Voters

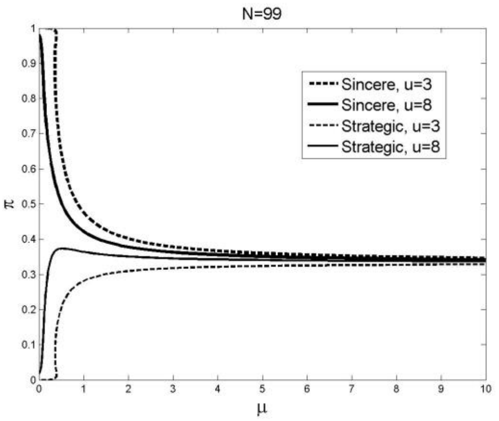

Notes. Lines show the principle branch of the MLC for high (u^m=8) and low (u^m=3) values of the intermediate option when (N_{ABC},N_{BCA},N_{CAB}) = (33,33,33).

As with the small voting body (N=12) reported in the main text, the results for these medium sized legislatures (N=99) are very comparable to the uninformed case. In the informed case, for $\mu\in$[0.4,0.8] the MLE probability of a strategic vote is approximately 0.38 when u^m=8 and increases from close to 0 to 0.26 for u^m=3.

[1] For example, error need not be equally likely for all options. Intuitively, it would seem that the utility from the dominated alternative winning is much less prone to noise than the utility of having the most favored option win. Similarly, there is no reason why noise in small voting bodies would have the same distribution as in large electorates or why would be equal across voters. Adapting the model in these ways could lead to other results than those presented here.

Appendix F - MLC per voter type

This appendix presents and discusses the MLC per voter type. To start, Figure F1 plots the weighted average of the Principal Branch of the MLC for these four sets.[2]

Figure F1: Strategic Voting by Voter Types

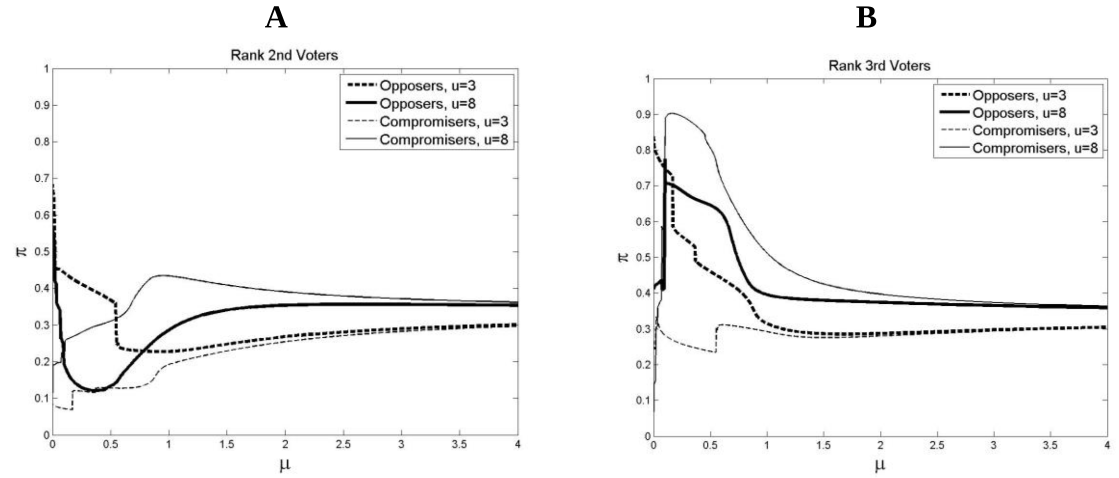

Notes. Lines show the weighted average of the principal branches of the MLCs, distinguishing between high (u^m=8) and low (u^m=3) values of the intermediate option in combination with a voter's Incentive-Type. Rank 2nd voters are shown in panel A and Rank 3rd voters in panel B. Only the equilibrium probabilities of voting strategically are shown. The average is across all possible combinations of preference orderings, weighted by the probabilities with which they occur. Cases where groups are tied for Rank 2nd are not included in the graph (*cf.* fn 24).

First note that different types play distinct strategies. In the Nash equilibrium (as $\mu\downarrow 0$) Opposers tend to vote strategically. When ranked 3rd with a low intermediate value, the Nash equilibrium probability is highest (almost 0.85). Irrespective of rank and intermediate value, Opposers vote more strategically than Compromisers in this limiting MLE.[3] With noise, in particular when $\mu\in[0.4,0.8]$, Rank 3rd voters vote mostly strategically in the MLE.[4] The Incentive-Type matters, however. When u^m=3, Rank 3rd voters are more likely to vote strategically if they are Opposers than if they are Compromisers. The reverse holds for u^m=8.

The latter result is in line with intuition. When they are Compromisers, Rank 3rd voters second choice is the Majoritarian Candidate. A strategic vote is likely to be successful because supporters of this candidate rarely vote strategically. For the high importance of the intermediate option, the benefits of a strategic vote are relatively high. When they are

[2] Separate graphs for each unique situation are available upon request.
[3] Not shown in the figure is that in the selected Nash equilibrium, supporters have relatively low probabilities of voting strategically (between 0.05 and 0.17).
[4] The exception is the group of Rank 3rd Compromisers facing low intermediate value. The MLE for this group is approximately 0.3 for these μ-values.

Compromisers, Rank 3rd voters are therefore likely to vote strategically. When they are Opposers, a strategic vote is an attempt to collaborate with the Rank 2nd voters, who themselves are Compromisers. The attraction of a strategic vote is diminished by the fact that the voters it supports are themselves inclined to vote strategically for the Majoritarian Candidate, decreasing the probability of success.

With a low intermediate value, the interpretation is more complex. First, note that in this case Rank 3rd voters vote less strategically anyway. When Rank 2nd voters are Compromisers, the appeal for a strategic vote is lower than with high intermediate value. Therefore, in equilibrium, they settle less for a compromise, creating a chance for the Rank 3rd voters (Opposers) to vote strategically by supporting the option most preferred by the Rank 2nd voters. When Rank 3rd voters are Compromisers, Rank 2nd voters are Opposers. A strategic vote by the latter means voting for the option most preferred by Rank 3rd. The incentive for Rank 3rd voters to compromise is them low, especially when together with Rank 2nd voters they have a strong majority. This reasoning implies an increased probability of a strategic vote by Rank 2nd voters (Opposers) and a decreased probability for Rank 3rd voters (Compromisers).

We use this analysis to derive behavioral prediction 4:

- With full information Rank 3rd voters vote more strategically (on average) than other Rank-Types (figure F1)

Moreover, we derived the following combined ***Rank-Type*** and ***Incentive-Type*** behavioral predictions:

- With full information and low value for the intermediate option Rank 3rd voters will more likely vote strategically if they are Opposers than if they are Compromisers (figure F1 B).
- With full information and low value for the intermediate option Rank 2nd voters will more likely vote strategically if they are Opposers than if they are Compromisers (figure F1 A).

Combined, they lead to behavioral prediction 5:

- With full information and low value for the intermediate option, Opposers are more likely to vote strategically than Compromisers.

Similarly, we derive the behavioral predictions:

- With full information and high value for the intermediate option Rank 3rd voters will more likely vote strategically if they are Compromisers than if they are Opposers (figure F1 B).
- With full information and high value for the intermediate option Rank 2nd voters more likely vote strategically if they are Compromisers than if they are Opposers (figure F1 A).

Combined, they lead to behavioral prediction 6:

- With full information and high value for the intermediate option, Compromisers are more likely to vote strategically than Opposers.

Appendix G - Experimental instructions

In this appendix we provide a transcript of the instructions for the treatment with high intermediate value and full information. The paragraph denoted in italics was omitted in the treatment without information. Note that there were 24 subjects (i.e., two independent electorates) in the laboratory in any given session.

Welcome

Welcome to this experiment in decision making. Please read these instructions carefully. They will explain the situations you will be facing and the decisions you will be asked to make.

In this experiment you will earn money, which will be paid to you privately at the end of the session. Your earnings will depend on your decisions as well as on the decisions of other participants in today's experiment.

Your earnings in the experiment will be in experimental "points". At the end of the experiment, each experimental point will be exchanged for euros at a rate of €0.05 per point. For example, if you earn 200 points, your earnings will be € 10. In addition, you have already received € 7 for showing up on time.

Rounds and Decisions

In this experiment, you will play various rounds. The total number of rounds will not be revealed, however. In each round, you will be asked to make exactly one decision.

Your decision in any round consists in voting for one of the options: A, B or C. The electorate consists of 12 people whose identities will not be revealed. This electorate will be kept fixed during the whole experiment. Each member of the electorate will have the same three options to vote from.

The option elected will be the one receiving the highest number of votes (out of 12). In case of a tie, one of the options with the highest number of votes will be randomly selected with equal chance.

Your Preference Ordering

In **each round** you will be assigned a preference ordering which will determine your earnings according to the winner of the vote.

Your preference ordering, and the preference ordering of your colleagues, can be one of the following:

A B C

B C A

C A B

In case the elected option is the option listed first you will receive **10 points**;
In case the elected option is the option listed second you will receive **8 points**;
In case the elected option is the option listed last you will receive **1 point**.

In **each round**, each of the 3 preference orderings will be attributed to **each person** independently with equal chance. Therefore, your preference ordering will often change from one round to another. Before you cast your vote, you will be informed of your preference ordering for that round. We advise that at the start of every round you take a moment to check this preference ordering.

In addition, at the start of every round, you will be informed how many participants in your electorate have been attributed to each of the three preference orderings. For example, you may hear that 5 voters have preference ordering A B C, 3 voters have B C A and 4 voters have C A B. In addition, you will also know your own preference ordering for the round, of course.

Trial Round

Before we start with the actual experiment, there will be one trial round at the start of the experiment. This trial round proceeds in exactly the same way as the rounds in the experiment itself, but it will have no consequences for actual earnings.

Appendix H - Preference distributions used

Table H1 shows the realizations of the random draws for the preference distributions for the 40 elections. The same realizations were used in all electorates.

Table H1: Realized Preference Distributions

Election	ABC	BCA	CAB	Majoritarian Set	Majoritarian Candidate
1	4	5	3	B	B
2	1	4	7	C	C
3	3	5	4	B	B
4	3	4	5	C	C
5	2	6	4	B	B
6	7	2	3	A	A
7	6	3	3	A	A
8	4	5	3	B	B
9	3	6	3	B	B
10	1	7	4	B	B
11	5	1	6	C	C
12	6	4	2	A	A
13	4	3	5	C	C
14	3	3	6	C	C
15	2	9	1	B	B
16	4	2	6	C	C
17	7	3	2	A	A
18	2	4	6	C	C
19	4	1	7	C	C
20	3	1	8	C	C
21	4	4	4	ABC	ABC
22	5	5	2	AB	A
23	2	5	5	BC	B
24	4	4	4	ABC	ABC
25	3	4	5	C	C
26	4	5	3	B	B
27	4	3	5	C	C
28	2	6	4	B	B
29	5	4	3	A	A
30	4	3	5	C	C
31	2	4	6	C	C
32	8	1	3	A	A
33	2	7	3	B	B
34	2	6	4	B	B
35	3	5	4	B	B
36	5	5	2	AB	A
37	10	2	0	A	A
38	5	1	6	C	C
39	2	2	8	C	C
40	5	5	2	AB	A
